# Role of Store-Operated Ca^2+^ Entry in the Pulmonary Vascular Remodeling Occurring in Pulmonary Arterial Hypertension

**DOI:** 10.3390/biom11121781

**Published:** 2021-11-27

**Authors:** Bastien Masson, David Montani, Marc Humbert, Véronique Capuano, Fabrice Antigny

**Affiliations:** 1 Faculté de Médecine, School of Medicine, Université Paris-Saclay, 94276 Le Kremlin-Bicêtre, France; bastienmasson999@gmail.com (B.M.); davidmontani@gmail.com (D.M.); mjc.humbert@gmail.com (M.H.); veronique.capuano@universite-paris-saclay.fr (V.C.); 2 INSERM UMR_S 999 Pulmonary Hypertension: Pathophysiology and Novel Therapies, Groupe Hospitalier Paris Saint-Joseph, Hôpital Marie Lannelongue, 92350 Le Plessis-Robinson, France; 3 Assistance Publique—Hôpitaux de Paris (AP-HP), Department of Respiratory and Intensive Care Medicine, Pulmonary Hypertension National Referral Center, Hôpital Bicêtre, 94276 Le Kremlin-Bicêtre, France; 4 Research and Innovation Unit, Groupe Hospitalier Paris Saint-Joseph, Hôpital Marie Lannelongue, 92350 Le Plessis-Robinson, France

**Keywords:** PAH, Ca^2+^ signaling, Orai, STIM, TRPC, IP_3_R, RyR

## Abstract

Pulmonary arterial hypertension (PAH) is a severe and multifactorial disease. PAH pathogenesis mostly involves pulmonary arterial endothelial and pulmonary arterial smooth muscle cell (PASMC) dysfunction, leading to alterations in pulmonary arterial tone and distal pulmonary vessel obstruction and remodeling. Unfortunately, current PAH therapies are not curative, and therapeutic approaches mostly target endothelial dysfunction, while PASMC dysfunction is under investigation. In PAH, modifications in intracellular Ca^2+^ homoeostasis could partly explain PASMC dysfunction. One of the most crucial actors regulating Ca^2+^ homeostasis is store-operated Ca^2+^ channels, which mediate store-operated Ca^2+^ entry (SOCE). This review focuses on the main actors of SOCE in human and experimental PASMC, their contribution to PAH pathogenesis, and their therapeutic potential in PAH.

## 1. Overview of SOCE

In mammals, the modulation of intracellular Ca^2+^ concentration ([Ca^2+^]_i_) constitutes a signal transduction mechanism for all cell types, regulating a large range of cellular functions, including contraction, proliferation, migration, gene transcription, metabolism, death, and apoptosis [1,2,3]. Intracellular Ca^2+^ signals are generated by Ca^2+^ release from the internal stores and/or by Ca^2+^ entry from the extracellular compartment through Ca^2+^ channels.

One of the major Ca^2+^ channels located in the plasma membrane (PM) is store-operated Ca^2+^ channels (SOCs), which produce store-operated Ca^2+^ entry (SOCE). The principle of SOCE was first described by Putney in 1986 [4] in non-excitable cells, where he found that extracellular Ca^2+^ influx was coupled to endoplasmic reticulum (ER) Ca^2+^ depletion, and then SOCE could be pumped into the ER by sarcoplasmic/endoplasmic reticulum Ca^2+−^ATPase (SERCA) to restore ER Ca^2+^ homoeostasis. A few years later, in mast cells, Hoth and Penner provided proof that SOCE is partly mediated by Ca^2+^ currents called Ca^2+^ release-activated Ca^2+^ (CRAC) currents (*I_CRAC_)* [5]. *I_CRAC_* is a non-voltage-activated Ca^2+^ inward current with strong inward rectification, a positive reverse potential, and a high selectivity for Ca^2+^ ions [5]. *I_CRAC_* is regulated through negative feedback that prevents excessive Ca^2+^ influx called Ca^2+^-dependent inactivation (CDI) [5,6]. CDI is composed of fast CDI and slow CDI.

Over the past several years, evidence from a large variety of cell types has demonstrated the existence of SOCE, and its involvement in cellular processes makes it a potential target for many diseases [7]. For two decades, the molecular identity of SOCs has been unknown. However, the advent of molecular biology techniques has made it possible to identify the molecules responsible for SOCE. In 2005–2006, two major ubiquitous SOCE actors were identified: the stromal interacting molecule (STIM) [8,9] and the Ca^2+^ channel Orai activated by STIM [10]. Additionally, in several cell types, in addition to Orai Ca^2+^ channel activation, STIM molecules activate transient receptor potential canonical channels (TRPCs), which are nonselective cation channels permeable to Ca^2+^.

### 1.1. STIM1

The link between ER Ca^2+^ depletion and Ca^2+^ entry was resolved with the discovery of STIM1 [8,9]. STIM1, a single pass transmembrane protein predominantly located in the ER, is able to sense luminal [Ca^2+^] and activate Orai and TRPC channels.

**Figure 1:**
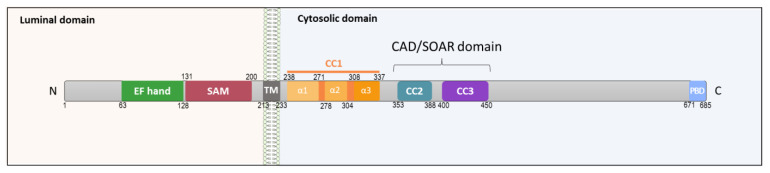
Topology and key domain organization of STIM1. A STIM1 monomer consists of an N-terminal luminal part, a transmembrane domain and a C-terminal cytosolic part. CC, coiled-coil; CAD, CRAC activation domain or SOAR, STIM–Orai activation region; EF hand, helix-loop structural domain; PBD, polybasic domain; SAM, sterile alpha motif; TM, transmembrane domain. Numbers correspond to the location of amino acid.

STIM1 is constituted by a luminal N-terminal part, which notably contains a Ca^2+^-binding EF hand domain that is able to sense the variation in the ER Ca^2+^ concentration ([Ca^2+^]_ER_) [11,12,13,14] (Figure 1). This EF hand domain forms with the sterile alpha motif (SAM) of the EF–SAM domain, which oligomerizes after ER Ca^2+^ depletion [12,15,16].

This luminal part is followed by a transmembrane segment (TM), potentially involved in the conformational change in STIM upon ER Ca^2+^ depletion [17]. The cytosolic part of STIM1 is composed of three coiled-coil parts (CC1, CC2, and CC3) (Figure 1). The structural interplay between these three CC domains is necessary for the elongation of STIM1 during its activation [18,19,20]. CC2 and CC3 domains are part of the STIM1–Orai1-activating region (SOAR) [21,22,23], also called CAD for CRAC-activating domain, and are necessary for coupling with Orai1 protein and for Orai1 activation [18,24]. SOAR is followed by a positively charged polylysine domain, which allows STIM1–TRPC electrostatic interaction [25]. The COOH cytosolic part ends in a polybasic domain (PBD), a domain required for the accumulation of STIM1 into puncta at the ER–PM junction by interaction of PBD with negatively charged phospholipids in the PM [22,26,27]. The STIM family is also composed of STIM2, which has high sequence homology with STIM1 with some differences at the N- and C-terminal parts [11]. STIM2 is more sensitive to [Ca^2+^]_ER_ changes due to its lower affinity for Ca^2+^ and needs a lower level of store depletion than STIM1 for its activation [28,29]. Here, we only described the mechanism of action of SOCE by STIM1 because it is better characterized than STIM2.

Over the years, few additional variants have been found for STIM1 and STIM2. STIM1 long (STIM1L) was described as the product of alternative splicing of exon 11. STIM1L forms permanent clusters that colocalize with Orai1 through an interaction between STIM1L and actin fibers via the additional actin-binding domain (not present in classical STIM1). Most likely, due to its preactivated form STIM1L, it has faster activation than STIM1 associated with rapid activation of SOCE, as demonstrated in skeletal muscle cells [30]. A neuron-specific splice variant called STIM1B was also found, which is specifically localized in the presynaptic ER, explained by a targeting motif added through splice insertion in the cytosolic region of STIM1. STIM1B slowly activates SOCE and reduces Ca^2+^-dependent inactivation of *I_CRAC_* [31]. For STIM2 variants, there are STIM2.1 (or STIM2β) and STIM2.3, considering that the classical STIM2 (known to activate SOCE) is called STIM2.2 or STIM2α. STIM2.1 is known to negatively regulate SOCE, probably through a sequence-specific allosteric interaction with Orai1 [32,33].

### 1.2. Orai Channels

In 2006, Feske et al. described the absence of *I_CRAC_* on T cells isolated from patients with severe combined immune deficiency (SCID) syndrome [34]. This loss of *I_CRAC_* was independent of Ca^2+^ stores or the expression of several genes implicated in the control of Ca^2+^ entry in lymphocytes (*KCNA3*/Kv1.3, *KCNN4*/IKCa1, *TRPC1*, *TRPC3*, *TRPV6*, *STIM1*). By sequencing genomic DNA from SCID patients, they identified loss-of-function mutations in *ORAI1*, identifying Orai1 as an essential archetype of the CRAC current [10]. At the same time, by genome-wide RNA interference screening in Drosophila S2 cells, Zhang et al. confirmed that *olf186-F* (named Orai1) was the main channel of I*_crac_* [35], which was subsequently confirmed by further studies [36,37,38].

**Figure 2:**
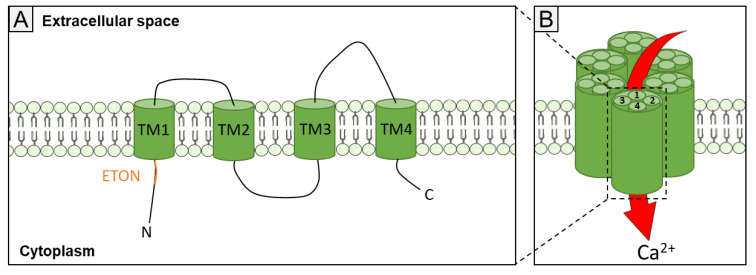
Orai1 topology and key domain for interaction with STIM1. (**A**) The Orai1 monomer consists of an ETON domain in the N-terminal part, four transmembrane domains (TM1-TM4) and a C-terminal domain. ETON, extended transmembrane Orai1 N-terminal. (**B**) Schematic assembly of Orai1 subunits as a hexamer.

The Orai1 channel presents a hexameric structure, as defined by the crystal structure of *Drosphilia melanogaster* Orai [39]. This architecture is formed by six Orai1 subunits coming from three dimers (Figure 2). Each of them contains an assembly of four transmembrane α-helices (TM1-TM4). This arrangement is made around a central point: the ion pore, with the 6 M1 subunits lining the ionic pore. The N- and C-termini are in the cytosol and contain the binding sites for the STIM protein, notably with the extended transmembrane Orai1 N-terminal (ETON) region. The ETON region mediates the interaction with the SOAR of STIM1 [40].

The Orai family consists of three members: Orai1, Orai2, and Orai3. All three members are widely expressed in humans [41] and can constitute functional CRAC channels [14,42,43]. Co-expression of STIM1 and Orai2 or Orai3 induced Ca^2+^ currents similar to those of Orai1-induced *I_CRAC_*, with small differences in selectivity and inactivation. Regarding selectivity, monovalent cations, such as Na^+^, permeate better in Orai3 than in Orai1 and Orai2 [44]. Regarding Orai inactivation, Orai2, and especially Orai3, have higher levels of fast CDI, and Orai2 does not have slow CDI [44,45].

In humans, Orai1 has two isoforms: the longer-form Orai1α, of approximately 33 kDa, and the shorter-form Orai1β, of approximately 23 kDa, resulting from an alternative translation initiation site [46]. Both isoforms can support SOCE [47].

### 1.3. TRPC Channels

TRPC channels are part of the transient receptor potential (TRP) channels, a family of cationic nonselective channels (Ca^2+^, Na^+^, K^+^). This group of channels is subdivided into seven distinct subfamilies according to their sequence homology [48,49]: TRPC (canonical), TRPV (vanilloid), TRPM (melastin), TRPA (ankyrin), TRPP (polycystin), TRPML (mucolipin), and TRPN (*drosophilia* no mechanoreceptor potential C (NOMPC)). Except for TRPM4 and TRPM5, all TRP channels are cationic channels permeable to Ca^2+^ [50]. In the present review, we only focused on TRPC channels because evidence from the literature indicated that TRPC channels are involved in SOCE.

The TRPC family can be divided into four subgroups: TRPC1; TRPC3, -C6, -C7; TRPC4, -C5; and TRPC2, which is a pseudogene in humans [51]. TRPC channels are mostly Ca^2+^- and Na^+^-selective, except TRPC2, which is cation-nonselective [52].

**Figure 3:**
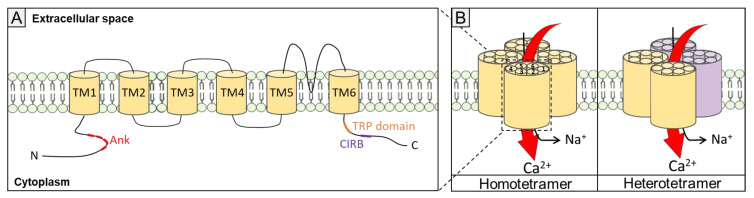
General topology and key domains of TRPC channels. (**A**) The TRPC subunit contains four to five ankyrin-like repeats on the N-terminal side, six transmembrane domains (TM1-TM6), a TRP domain and a CIRB domain on the C-terminal side. Ank, ankyrin-like repeats; CIRB, calmodulin–IP3R binding site. (**B**) Schematic assembly of TRPC subunits as a homotetramer or heterotetramer.

Structurally, TRPC channels are relatively analogous [53,54]. They can form heterotetramers or homotetramers. Each TRPC channel consists of at least six transmembrane domains with an ionic pore formed between TM5 and TM6 (Figure 3). On the N-terminal cytosolic domain, there are four to five ankyrin repeats, leading to protein–protein interactions [55,56]. On the C-terminal side, the TRP domain, a well-conserved region in all TRPCs, is essential for channel gating [54], followed by the calmodulin (CaM)/inositol 1,4,5-trisphosphate receptor-binding (CIRB) site, with CaM binding in a Ca^2+^-dependent manner [51,57,58]. Interestingly, incorrect trafficking of TRPC3 was observed when the CIRB region was deleted [59].

TRPC channels are reported to contribute to both SOCE and receptor-activated Ca^2+^ entry pathways (ROCE). As described below, several studies have demonstrated TRPC channel activation following ER Ca^2+^ depletion.

TRPC1 was first reported to have a role in SOCE from studies using the expression of the splice variant of TRPC1, so-called TRPC1A, in heterologous cell system expression [60] or the expression of a full-length cDNA of human TRPC1 in COS (fibroblast-like cell lines derived from monkey kidney tissue) cells [61]. The role of TRPC1 was confirmed through studies using different approaches (RNA interference, dominant-negative overexpression) in human submandibular gland cell lines [62], endothelial cells [63], human myoblasts and myotubes [64,65,66], and platelets [67]. Further evidence was provided by studies on *trpc1*^−/−^ mice that showed reduced SOCE in different tissues (salivar gland, pancreatic acinar cells, and aortic endothelial cells) [68,69,70].

TRPC3 was found to be activated by depletion of Ca^2+^ stores in DT40 chicken B lymphocytes [71]. Selective suppression of TRPC3 protein by small interfering RNA (siRNA) showed TRPC3-mediated SOCE in H19-7 hippocampal neuronal cells [72] and human umbilical vein endothelial cells [73]. When TRPC3 is expressed at low levels in HEK293 cells, the TRPC3 current is inhibited in cells treated with siRNA against STIM1, showing the regulation of TRPC3 by STIM1 [74]. Salivary glands and pancreatic cells isolated from *trpc3^−/−^* mice and cells treated with a TRPC3 inhibitor exhibit reduced SOCE [75,76]. The regulatory mechanism of TRPC3 appears to be determined by its expression level [71].

TRPC4 has been shown to be activated by Ca^2+^ store depletion in HEK293 cells [74] and *Xenopus laevis* oocytes [77]. Other evidence has been obtained notably by siRNA strategy in human myoblasts and myotubes [64,65], human adrenal cells [78], human corneal endothelial cells [79], human gingival keratinocytes [80], neonatal rat ventricular cardiomyocytes [81] or human and mouse endothelial cells [82]. TRPC4 implication in SOCE has also been shown in neonatal rat ventricular cardiomyocytes by a dominant-negative strategy [81]. In addition, aortic and lung endothelial cells from *trpc4^−/−^* mice showed reduced SOCE [83,84].

Few studies report the implication of TRPC5 in SOCE, although it was found in smooth muscle cells isolated from rabbit pial arterioles [85] and in neonatal rat ventricular myocytes [81,86].

Regarding TRPC6, several studies have shown that it is also able to drive SOCE. This was shown by siRNA in a leukemic granulocyte cell line [87], antibody inhibition in human platelets [88,89,90], or overexpression and knockdown by short hairpin RNA (shRNA) in hepatoma cells [91]. On the other hand, in HEK293 cells co-transfected with STIM1 and TRPC6, STIM1 does not influence the TRPC6-induced current [92], except when TRPC6 is expressed at low levels [74].

TRPC7 is not regulated by STIM1 or Orai1, and it was proposed that TRPC7 is activated by a mechanism independent of store depletion [93].

It is also known that some TRPC channels can be directly activated by the phospholipase C (PLC) pathway, notably with the PI(4,5)P2 degradation product diacylglycerol (DAG), by the G protein coupled receptor, protein kinase C (PKC) pathway, or even membrane stretching [73,94,95], resulting in receptor-operated Ca^2+^ entry (ROCE) instead of SOCE.

**Figure 4:**
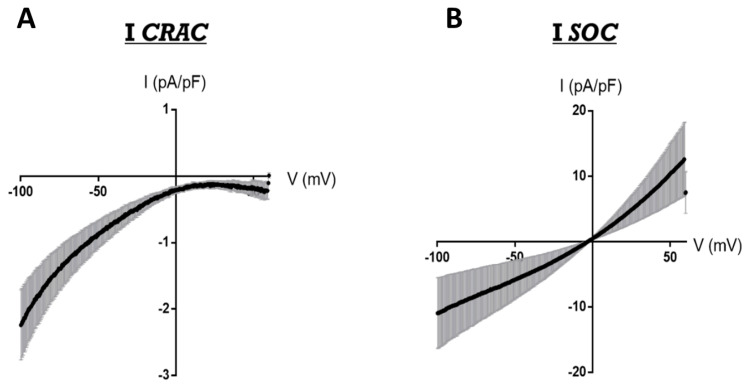
Comparison between ICRAC and ISOC in HEK cells transfected with STIM1 + Orai1. Cells were transfected with Orai1 and STIM1 plasmids 24 h before electrophysiological recordings. (**A**) Representative *I_CRAC_* produced by STIM1+Orai1, inward rectifier current with almost no inward potential. In more than 60% of cells displaying *I_CRAC_*. (**B**) Representative *I_SOC_* produced by mixing STIM1+Orai1 and endogenous TRPC channels (linear current with inward potential close to 0 mV). In almost 40% of cells displaying *I_SOC_*. Patch-clamp recordings were performed in whole-cell mode using electrodes of resistance between 2.5–3 MΩ. The pipette solution contained (in mM) 130 caesium methansulfonate, 8 MgCl2, 10 HEPES, 10 BAPTA, and 0.001 thapsigargin, pH 7.2 (CsOH). Extracellular solution contained (in mM): 135 NaCl, 10 CsCl, 4.4 MgCl2, 2.8 KCl, 10 Hepes, 0.5 EDTA, 0.5 EGTA, 10 glucose, and 11 CaCl2, pH 7.4 (NaOH). After measuring the maximal current amplitude, 10 μM Gd^3+^ was added at the end of the recording to block the current and estimate the leak. Currents were recorded during a 1 s ramp of potentials ranging from −130 mV to +85 mV applied with low-pass filtering at 1 kHz.

However, due to their electrophysiological characteristics (Table 1), TRPC channels are not able to produce *I_CRAC_*. Indeed, TRPC channels form nonselective Ca^2+^-permeable channels that do not exhibit the electrophysiological identity of *I_CRAC_* (Figure 4A). However, almost all TRPC channels are activated in response to ER–SR Ca^2+^ depletion, with the formation of a nonselective current with a reverse potential close to 0 mV, called *I_SOC_* (Figure 4B).

### 1.4. SOCE Mechanism

The activation mechanisms leading to SOCE at the PM by STIM proteins are now better understood: one of the most physiological mechanisms inducing ER–SR Ca^2+^ store depletion is the activation of the PLC via G-protein coupled receptors, with inositol 1,4,5-triphosphate (IP3) generation and IP3 receptor (IP3R) activation [107,108]. In cells with furnished Ca^2+^ stores, STIM1 maintains an inactivated conformation and localizes throughout the ER–SR membrane. In this conformation, STIM1 is able to bind Ca^2+^ with his EF hand–SAM domain. This inactivated conformation of STIM1 prevents spontaneous activation since the CC1 domain is associated with CAD–SOAR [17,109,110].

**Figure 5:**
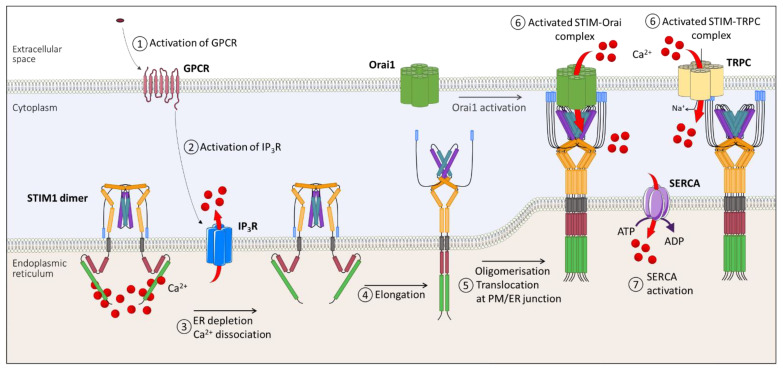
Activation mechanism of store-operated Ca^2+^ channels. Depletion of intraluminal Ca^2+^ concentration triggers Ca^2+^ dissociation of the EF hand of STIM1, allowing elongation and oligomerization of STIM1 at the ER–PM junction. This allows the interaction of STIM1 with Orai1 and/or TRPC channels, allowing Ca^2+^ entry into the cytoplasm. ADP, adenosine diphosphate; ATP, adenosine triphosphate; ER, endoplasmic reticulum; GPCR, G-protein coupled receptor; IP_3_R, inositol 1,4,5-trisphosphate receptor; PM, plasma membrane; SERCA, sarcoplasmic/endoplasmic reticulum Ca^2+−^ATPase; TRPC, transient receptor potential canonical channels.

After ER–SR Ca^2+^ depletion, the luminal side of STIM1 rearranges by oligomerization of the EF–SAM domain [12,15,16] (Figure 5). This association induces the modification of the TM domain, leading to the liberation of CAD–SOAR [20]. This conformational change leads to rapid oligomerization of STIM1 through the CC3 domain and, thus, translocation to the ER–PM junction [18,26,111]. After this oligomerization and translocation, STIM1 forms puncta in this region where Orai is also located. It was shown that one to two STIM1 channels could trap Orai1, but eight STIM1 channels for each Orai1 channel were required to optimally activate these channels [112]. STIM1 interacts and activates with the ETON domain of Orai1 via CAD–SOAR^97^. The Ca^2+^ stores of the ER–SR will then fill up through the activity of the SERCA pump located on the ER–SR membrane. I*_crac_* is then regulated through the CDI.

It has been shown that STIM1 can regulate all TRPC channels, except TRPC7 [74]. STIM1 can bind TRPC1, -C4, and -C5 [74,113] via electrostatic interactions that exist between two positively charged lysins (^684^KK^685^) on STIM1 and two negatively charged conserved aspartates (^639^DD^640^) on TRPC1 [25]. Such electrostatic interactions also seem to occur with TRPC4 and TRPC5 [114]. Alternatively, STIM1 can regulate TRPC3 and TRPC6 indirectly through heteromultimerisation of TRPC1 with TRPC3 and TRPC4 with TRPC6 [74]. Furthermore, it has been demonstrated that local Ca^2+^ entry via Orai1 channels can regulate plasma membrane recruitment of TRPC1 [115]. TRPC1-containing vesicles can be detected in the region of the subplasma membrane in the immediate vicinity of the ER–PM region where Orai1 and STIM1 aggregate upon Ca^2+^ depletion. Recruitment of TRPC1 for clustering with STIM1 in the ER–PM junction occurs by trafficking via fast recycling endosomes [116]. Despite these studies and putative models, the precise mechanism of TRPC channel contributions in SOCE remains to be elucidated.

In addition to SOCE, activation of PLC-coupled receptors can activate the store-independent Ca^2+^ entry (SICE) pathway. The SICE pathway is performed through arachidonic acid-regulated Ca^2+^-selective (ARC) [83,117] and leukotriene C_4_ (LTC_4_)-regulated Ca^2+^ (LRC) [118]. These channels are composed of both Orai1 and Orai3 subunits [119,120] and can be regulated by a pool of STIM1 at the PM [119,121], notably through the interaction of STIM1 with Orai3 [122].

## 2. SOCE in PAH

### 2.1. Pulmonary Arterial Hypertension

Pulmonary arterial hypertension (PAH) is a progressive and devastating disorder due to a progressive obstruction of distal pulmonary arteries (PA) (<500 μm in diameter), resulting in an elevation of pulmonary arterial pressure and progressive right heart dysfunction [123]. Recently, PAH was proposed to be defined by a mean pulmonary arterial pressure (mPAP) higher than 20 mmHg (compared to a normal PAPm, a rest of 14 ± 3 mmHg), a pulmonary vascular resistance higher than 3 woods units and a pulmonary arterial wedge pressure lower than 15 mmHg [124,125].

PAH is clinically classified, not according to clinical manifestations but according to etiology. One of the most commonly diagnosed groups is idiopathic PAH (iPAH), which includes patients with no identified causative factor. PAH can also be heritable, induced by drugs and toxins, or associated with other diseases [125]. The classification also includes a subgroup for long-term responders to Ca^2+^ channel blockers, another one for PAH with overt features of venous capillary (pulmonary veno-occlusive disease or pulmonary capillary haemangiomatosis) involvement and a last one for persistent PH of the new-born syndrome [124].

PAH is characterized by an increase in pulmonary vascular resistance explained by endothelial dysfunction, excessive proliferation of pulmonary arterial smooth muscle cell (PASMC) and endothelial cell (PAEC), PA vasoconstriction, and in situ thrombosis [123]. PAEC dysfunction in PAH is mostly targeted by the existing three current therapies. The three main treatments target the prostacyclin pathway with prostacyclin analogues, the endothelin-1 (ET-1) pathway with ET-1 receptor antagonist and the nitric oxide (NO) pathway with PDE5 inhibitors or guanylate cyclase stimulators [126]. However, these treatments cannot cure PAH, and lung transplantation is still necessary for the most severe patients. As the prognosis remains poor with a 5-year survival rate of approximately 60%, there is a need for innovative treatments to target pulmonary vascular remodeling [127,128].

SOCs are ubiquitous, and their role in PAEC dysfunction and right ventricular (RV) failure occurring in PAH should be studied with interest. Indeed, SOCs contribute to systemic PAEC proliferation and migration [73,129] and right ventricular Ca^2+^ handling remodeling in experimental PH models [130,131].

Moreover, PASMC dysfunction is not directly targeted by current PAH therapies. Therefore, we focused this review on the role of SOCE in PASMC dysfunction. PASMC dysfunction is characterized by excessive proliferation, apoptosis resistance, abnormal migration, and vasoconstriction [123,132], which strongly contribute to pulmonary artery remodeling. Since an increase in PASMC [Ca^2+^]_i_ is a key trigger of these biological processes, resulting in pulmonary vascular remodeling. SOC modification could act on pulmonary remodeling in PAH and, thus, emerge as a potential additional therapeutic target in PAH.

The understanding of PAH pathogenesis mainly emanates from an experimental model of PAH. Although they do not perfectly reproduce all the clinical parameters of PAH, they are considered PH animal models, mostly performed in rodents, with three main experimental procedures: monocrotaline (MCT), chronic hypoxia (CH), and Sugen 5416 hypoxia (SU/Hx).

### 2.2. Physiological Implication of SOCE in Control PASMC

The expression of Orai1/2/3 and STIM1/2 was detected in mouse, rat, and human PASMC (hPASMC) [133,134,135]. In hPASMC, Orai1 and STIM1/2 promotes SOCE [136,137]. In rodent PASMC, Orai1/2/3 and STIM1/2 promote SOCE [134,135,138,139,140]. Only TRPC1, -C3, -C4, and -C6 were expressed at the mRNA and protein levels in primary hPASMC cultures [141,142,143,144,145,146], while TRPC1, -C3, -C4, -C5, and -C6 were expressed in rodent PASMC (Table 2).

TRPC1 is overexpressed in proliferative control hPASMC in association with increased SOCE, indicating that TRPC1 contributes to SOCE in control hPASMC [143,157]. In systemic vascular smooth muscle cell (VSMC), caveolin-1 knockdown by siRNA induces a decrease in TRPC1 expression accompanied by a reduction in SOCE [158]. Furthermore, in mouse PASMC, STIM1 and TRPC1 are functionally coupled and mediate SOCE [159].

The inhibition of TRPC1 by a specific antibody or by the nonselective SOCE inhibitor 2-aminoethyldiphenylbonirate (2-APB) significantly reduced ex vivo neointimal growth in human veins as well as SOCE and proliferation of cultured PASMC. These results highlight the involvement of TRPC1 in vascular remodeling occurring in arteriosclerosis and the protective potential of TRPC1 inhibitors against vascular disease [160].

STIM1 mediates the proliferation of primary human coronary artery SMC [161]. Moreover, the knockdown of STIM1 or Orai1 reduced SOCE and the migration of human saphenous VSMC. Alternatively, the overexpression of the negative form of TRPC5 reduced cell proliferation and cell migration [162]. In addition, an Orai1 blocker, Synta66 (S66), reduced PDGF-evoked human saphenous VSMC migration without a significant change in cell proliferation or viability.

In cultured rat aortic VSMC, Orai1 and STIM1 are overexpressed compared to freshly isolated VSMC, suggesting that these proteins enhance the VSMC proliferative phenotype [163]. STIM1, Orai1, and TRPC1 expression/function are crucial for angiotensin II- or urotensin II-induced rat VSMC proliferation [164,165].

Moreover, PASMCs have two phenotypes: a contractile phenotype, accessible in freshly isolated cells, and a proliferative and migratory phenotype, generally obtained in culture or pathological status. Treatment of rat proliferative PASMC with factors (transforming growth factor β (TGF-β) and heparin), favoring the differentiation of PASMC into a contractile phenotype, enhanced SOCE. Indeed, TGF-β decreased Orai1/2, STIM2, and TRPC6 expression, and heparin decreased Orai1/2/3, STIM1/2, and TRPC6 expression [135].

In rat aortic SMC, STIM1 or Orai1 knockdown reduces SOCE and migration, while the knockdown of Orai2 or STIM2 has no effect on SOCE, proliferation, and migration [163]. In cultured rat aortic SMC, STIM1–Orai1-mediated Ca^2+^ influx plays a key role in platelet-derived growth factor (PDGF)-induced migration, since siRNA knockdown inhibits PDGF-induced migration [166]. Moreover, enhancement of Orai1 function with IA65, a pharmacological activator, results in an increase in rat VSMC migration [167].

These results indicate that Orai1-induced SOCE contributes significantly to in vitro systemic VSMC migration and proliferation. We hypothesize that the SOCE archetype Orai1 could contribute to aberrant PASMC migration and proliferation phenotypes in PAH.

In rat coronary arteries, silencing of Orai1 or STIM1 prevents urotensin-II-induced vasoconstriction [165]. In mouse aortae, VSMC-specific knockout of the *stim1* gene impaired SOCE and reduced the phenylephrine-induced vasoconstrictive response [168]. Moreover, the overexpression of TRPC1 enhances rat PA vasoconstriction [142]. These results indicate that SOCE could contribute to VSMC contraction. Similar to systemic vessels, we hypothesize that SOCs contribute to PA contraction as well as PA vasoconstriction in PAH. However, the contribution of SOCs to the regulation of pulmonary arterial tone has yet to be understudied under physiological conditions and in the physiopathological condition of PAH. In systemic VSMC, SOCE seems to be constituted by STIM–Orai and the TRPC channel complex [169,170]. In addition to their role in SOCE, the Orai–TRPC complex has been demonstrated to also regulate Ca^2+^-sensitive K^+^ channels (KCa), contributing indirectly to the regulation of resting membrane potential. In isolated rat aortic SMC, TRPC1 forms a complex with the large conductance KCa (BK_Ca_) that promotes Ca^2+^ entry through and then membrane hyperpolarization. This membrane hyperpolarization mediated by TRPC1–BK_Ca_ coupling could prevent excessive contraction of VSMC by reducing agonist-induced membrane depolarization [171].

### 2.3. Physiopathological Implication of SOCE in PAH PASMC

Several studies found an increase in SOCE in hPASMC isolated from iPAH patients, resulting in an increase in [Ca^2+^]_i_ [157,172]. Control hPASMC exposed to hypoxia (3% O_2_ for 72 h) saw an increase in [Ca^2+^]_i_ associated with an increase in Orai1/2, STIM1/2, and TRPC6 protein levels [173]. The knockdown of HIF-1α in rat PASMC or HIF-1α deficiency in mice (*hif-1α*^−/−^*)* reduced the overexpression of Orai2 induced by hypoxia exposure (10% O_2_, 21 days for mice or 4% O_2_, 60 h for rat PASMC) [134]. As the increased expression of HIF-1α plays a key role in the pathogenesis of PAH [174,175,176], we hypothesized that HIF-1α overexpression in PAH promotes an increase in SOCE in iPAH PASMC.

An increase in STIM2 protein levels has been demonstrated in iPAH hPASMC compared with control PASMCs, while the STIM1 protein level remained unchanged. Using a siRNA strategy, they also found that the knockdown of STIM2 reduces SOCE and proliferation of iPAH hPASMC without any effect on control hPASMC [136]. In an additional study, they demonstrated that the transition of rat PASMCs from a contractile to proliferative phenotype was associated with enhanced SOCE due to increased expression of Orai2, STIM2, and TRPC6 [135]. Moreover, they demonstrated that nicotinamide phosphoribosyl transferase promotes pulmonary vascular remodeling in PAH and experimental PH, partly due to an overexpression of Orai2 and STIM2 protein levels leading to an increase in SOCE and in hPASMC proliferation [177].

SOCE increased in rat distal PASMCs exposed ex vivo to acute hypoxia (4% O_2_) compared to rat proximal PASMC, in association with an increased expression of TRPC1 and STIM1 [178]. Another study also found increased expression of STIM1 in the distal PA of experimental PH in rats exposed to hypobaric chronic hypoxia CH (for 21 days) [140]. Additionally, in rat PASMC, it was demonstrated that the knockdown of STIM1 inhibited hypoxia-induced nuclear factor of activated T-cells (NFAT) cytoplasmic 3 (NFATc3) nuclear translocation and, thus, excessive PASMC proliferation [140]. In contrast to these studies, STIM1 expression was unchanged in rat distal PA and PASMC exposed to CH (10% O_2_ for 21 days) [134]. In this study, the authors found increased expression of Orai1/2, while Orai3 remained stable. It was also reported in mouse PASMC exposed to acute hypoxia (95% N_2_ and 5% CO_2_) that there was an increase in TRPC1, Orai1, and STIM1 interaction to mediate increased SOCE [151].

STIM1 and Orai1 expression and function were increased in aortas from spontaneously hypertensive rats [179]. In an experimental obstructive sleep apnea model induced in rats by chronic intermittent hypoxia (CIH) (5% inspired O_2_ for 20 s, followed by 280 s of room air, 12 times per hour for 8 h a day for 14 to 28 days), Castillo-Galán et al. showed the increased protein expression of Orai1, STIM1, and TRPC1, -C4, -C6 in the lungs of CIH rats [180]. Treatment of these rats with 2-APB decreased right ventricular systolic pressure (RVSP) and pulmonary vessel remodeling, which increased following CIH exposure [181].

In control hPASMC, platelet-derived growth factor (PDGF) application stimulated STIM1 and Orai1 overexpression through the AKT/mTOR signaling pathway, which led to an increase in SOCE [137]. As it is well recognized that PDGF is overproduced in PAH [182,183,184], this overproduction could explain why SOCE increased in iPAH hPASMC.

In experimental PH induced by CH exposure in mice, *trpc1*^−/−^ + *trpc6*^−/−^ double knockout protected mice against PH. In this experimental PH model, the deletion of *trpc1* and *trpc6* prevented pulmonary vessel remodeling after CH [152], demonstrating that TRPC1 and TRPC6 are essential for the development of PH under CH exposure in mice. In addition, Sun et al. showed that, in a CH-induced PH murine model (11% O_2_ for 28 days), intratracheal in vivo administration of siRNA against TRPC1 attenuates pulmonary and RV arterial remodeling, with a significant reduction in right ventricular systolic pressure [185]. In vivo TRPC1-siRNA delivery also counteracts the increase in inflammatory biomarkers (TNF-α and MMP-9) in the lung and apoptotic biomarkers in the RV myocardium that was observed in a murine model of CH-induced PAH.

In PH rats induced by CH exposure, TRPC1 and -C6 expression was increased in isolated PA, which was accompanied by an increase in SOCE. In addition, nonselective pharmacological inhibition of SOCE by lanthanum (La^3+^) and SKF-96365 normalizes elevated [Ca^2+^]_i_ in PASMC and vascular tone in PAs of CH–PH rats [145].

Another study found that TRPC1 mRNA levels were increased in murine PASMC under hypoxia exposure (1% O_2_ for 72 h). In this study, hypoxia exposure enhanced the proliferation of PASMC, which was reduced by the knockdown of TRPC1 (siRNA approach). The proliferation of PASMC isolated from *trpc1*^−/−^ mice was also reduced compared to the proliferation of PASMC isolated from *trpc1*^+/+^ mice. Furthermore, *trpc1*^−/−^ mice developed partial protection against PH in response to CH compared to *trpc1*^+/+^ mice exposed to CH. Both *trpc1*^+/+^ and *trpc1*^−/−^ mice exposed to CH present similar RV hypertrophy, while pulmonary vascular remodeling is attenuated in *trpc1*^−/−^ mice [153].

Increased TRPC1 and TRPC4 mRNA expression and protein levels in PAs isolated from MCT-PH rats were also found to be associated with increased SOCE and PA vasoconstriction [186].

Additionally, an increase in the expression of TRPC3 was described in iPAH hPASMC, which could partly explain the exacerbated SOCE in iPAH hPASMC^173^.

Using *trpc4*^−/−^ rats exposed to the sugen–hypoxia protocol (single subcutaneous injection of 20 mg/kg SU5416 followed by 10% O_2_ for 3 weeks), Alzoubi et al. found a reduction in the occlusion of small PA and plexiform lesions in *trpc4*^−/−^ rats compared with wild-type (WT) rats. However, *trpc4*^−/−^ rats have similar hemodynamic parameters to WT rats, but TRPC4 deficiency provided a significant survival benefit, with preservation of cardiac output [187].

Regarding the role of TRPC6 in PAH, single nucleotide polymorphisms (SNPs) were found in the promoter of the *TRPC6* gene in a few iPAH patients, facilitating the expression of the TRPC6 protein and increasing its function in iPAH [188,189]. Therefore, this SNP could predispose individuals who have this mutation to develop PAH.

TRPC6 expression (mRNA and protein) was found to be increased in lung and hPASMC from PAH patients, and the knockdown of TRPC6 by siRNA reduced the hyperproliferative phenotype of PAH hPASMC [146]. The role of TRPC6 was also predicted in PASMCs from Milan hypertensive strain (MHS) rats, a genetic model of systemic hypertension. Increased expression of TRPC6, SERCA2, and Na^+^/Ca^2+^ exchanger-1 (NCX1) was reported to likely contribute to the abnormal Ca^2+^ homoeostasis and subsequent vasoconstriction and increase blood pressure observed in MHS rats [190].

Recently, Jain et al. demonstrated that oral gavage with TRPC6 blocker BI-749237 reduced the development of PH induced by CH exposure in mice [191], suggesting that TRPC6 could be a potential target in PAH.

The majority of patients with heritable PAH have mutations in bone morphogenetic protein type 2 receptor (*BMPR2*) gene [192,193]. Reduction in BMPR-II signaling is a hallmark of PAH [194,195,196]. BMPR2 is activated by BMP ligands, including BMP2 and BMP4 [197]. BMP2 is protective against CH-induced PH, while BMP4 contributes to the development of PH [198]. BMP2 application in rat distal PASMCs induced a decrease in TRPC1, -C4, and -C6 expression (mRNA and protein levels), and therefore a reduction in SOCE, a reduction in basal [Ca^2+^]_i_, and a reduction in PASMC proliferation and migration [199]. Moreover, BMP4 application promotes pulmonary vascular remodeling in CH-PH mice, and *bmp4*^+/−^ mice exposed to CH were protected against the development of PH [198]. In rat distal PASMC, in contrast to BMP2 application, BMP4 application increases the expression of TRPC1, -C4, and -C6 (mRNA and protein levels); increases SOCE and [Ca^2+^]_i_; enhances PASMC proliferation and migration; and protects PASMC mice from apoptosis [154,155].

The resistance to apoptosis of hPASMC is also a hallmark of PAH. Currently, there are no studies on PAH hPASMC linking apoptosis resistance to SOCE. However, several studies have shown the role of Orai in the regulation of apoptosis in other cell types and diseases. For example, reduced Orai1 and STIM1 activity increases apoptosis of pancreatic cancer cells, and the in vivo silencing of Orai1 in mice protects macrophages from apoptosis [200,201]. On the other hand, it was demonstrated in human lymphoma B cell lines that pharmacological inhibition or silencing of Orai1 leads to an enhancement of apoptosis [202]. Thus, the contribution of SOCE and SOCs to the regulation of apoptosis resistance in iPAH PASMC needs to be further investigated.

The involvement of SOCs in hypoxic pulmonary vasoconstriction was also documented in rats using nonselective pharmacological approaches [203,204]. The real contribution of SOCs to the regulation of pulmonary arterial tone needs to be investigated using more selective pharmacology or genetic tools. The physiopathological implications of SOCs in PAH PASMC are summarized in Table 3.

Since SOCE contribute to the regulation of SR Ca^2+^ content and since SOCE is increased in PAH hPASMC, we can hypothesize that SOCE-induced increase in SR Ca^2+^ content result in an increase in the activity SR Ca^2+^ channels such as IP_3_R and ryanodine receptors (RyR). VSMC including PASMC exhibit three different RyR isoforms (RyR_1_, RyR_2_, and RyR_3_) and three different IP_3_R isoforms (IP_3_R_1_, IP_3_R_2_, and IP_3_R_3_) which could contribute to the regulation of PASMC functions. It has been demonstrated that IP_3_R_2_^−/−^ mice developed more severe PH than WT mice under CH-exposure [205]. Using isolated PASMC, Shibata et al. found that SOCE is strongly increased in IP_3_R_2_^−/−^ PASMC in normoxic condition as well as in the CH condition. The authors demonstrated that IP_3_R_2_ negatively regulated SOCE in mouse PASMC, indicating the presence of IP_3_R_2_ to protect against the development of experimental PH by regulating SOCE [205]. The contribution of the IP_3_R channel has not been studied in the aberrant phenotypes of PAH PASMC (proliferation, resistance to apoptosis, migration, contraction). IP_3_R was previously found to be functionally coupled with TRPC by a direct binding with the N-terminus of the IP_3_R and the C-terminal CIRB motif of the TRPC subtypes [57,206]. This direct binding with IP_3_R and TRPC has been demonstrated for all IP_3_R isoforms and all TRPC subtypes [58,207]. However, the mechanism by which IP_3_R isoforms regulate of TRPC function has not been yet demonstrated in PASMC from PAH patients.

In *ryr_2_*^−/−^mice, it has been demonstrated that an RyR_2_-mediated Ca^2+^ release contributes to pathological ROS generation induced by CH exposure [208]. SMC-specific *ryr_2_*^−/−^ mice have similar PAP in response to hypoxia compared to WT; however, RyR_2_ was suggested to have a role in the sustained phase of hypoxic pulmonary vasoconstriction [209]. In PASMC isolated from CH-PH and MCT-PH rats, important changes in the cellular localization of RyR isoforms and Ca^2+^ stores have been demonstrated, contributing to the pathogenesis of experimental PH [210]. The expression of TRPV4 and RyR_2_ proteins increased in isolated PA from CH-PH rats. In this study, the authors demonstrated that RyR_2_ regulated TRPV4 function in PASMC from CH-PH rats, contributing to excessive PA constriction [211].

## 3. Targeted SOCE in PAH: A Novel Therapeutic Option?

In PAH, the only ion channels targeted in the clinic are L-type voltage-gated Ca^2+^ channels. However, L-type voltage-gated Ca^2+^ channel blockers (nifedipine, diltiazem, or amlodipine) are only effective for less than 10% of PAH patients, named “the responder”, and defined by a fall of 10–40 mmHg in PAPm during inhalation of NO. Voltage-gated Ca^2+^ channel blockers act on the reduction in excessive pulmonary arterial vasoconstriction [212,213].

As SOCs constitute another important source of Ca^2+^ mostly involved in pulmonary artery remodeling, and L-type voltage-dependent Ca^2+^ channels are mainly involved in pulmonary artery vasoconstriction, targeting SOCs may be the next challenge to overcome PAH phenotypes. Indeed, PAH hPASMC are characterized by excessive proliferation, excessive migration, and vasoconstrictive phenotypes resulting from Ca^2+^ homoeostasis remodeling (particularly due to SOC activities).

Drugs acting directly and selectively on different SOCs could be attractive for improving PASMC remodeling in the context of PAH. As represented in Table 4, several compounds are available to differentially inhibit almost all SOCs.

Concerning inhibitors of Orai1, there is a well-known family of pyrazole derivatives, with the widely used BTP2 (YM-58483) [214,215,216,217,218] and GSK compounds (GSK7975A and GSK-5503A) [220], acting on the selectivity filter of Orai1, Synta-66, which binds directly to Orai1 [221,222,223,224] and its suitable in vivo analogue JPIII [225]. The lanthanide family (Gd^3+^ or La^3+^) has been known as an Orai blocker for many years [219,226]. The less-used AnCoA4 [227] and 5J4 [228] also exist. ML-9 is used to inhibit STIM1 puncta formation [229]. Since 2006 and the identification of Orai1 as the archetype of SOCE, Orai1 has been a potential target in several pathological processes, including psoriasis, pancreatitis, asthma, and COVID-19 pneumonia.

Auxora (or CM2489), an Orai1 channel inhibitor, was tested in a clinical trial to treat moderate to severe plaque psoriasis. However, it showed only a limited clinical effect but was safe and well tolerated by patients. The compound CM2489 had some chemical improvements and design to treat acute pancreatitis. The preclinical trials were successful in mice, and the phase I clinical trials were successful, allowing entry into phase II with a good safety profile and improvement in patient outcomes [245,246]. In addition, a clinical study, although limited by its design, has been conducted for the treatment of severe or critical COVID-19 pneumonia with this compound [247]. Another Orai1 inhibitor (RP3128) has reached clinical trials (Phase I/IIa) for asthma [248]. Finally, a last candidate, the Orai1 inhibitor PRCL-02, has achieved a phase IIa clinical trial for the treatment of moderate to severe chronic plaque psoriasis [249].

Concerning TRPC channel inhibitors used in experimental research, very few of them are selective because of the structural similarity between all TRPC channels. Xanthine-based inhibitors are known to inhibit TRPC1, -C5 and -C6 at nanomolar concentrations [232]. Among the xanthine-based inhibitors, HC-608 (also called Pico145), described to be one of the most potent inhibitors of TRPC1, -C4, and -C5 [234], and HC-070, is widely used to inhibit TRPC4 and -C5 [233]. The benzamidiole ML-204 is used for TRPC4 [231] and AC1903 for TRPC5 [235], but they both also inhibit TRPC4 and -C5. Alternatively, GFB-8438, produced from pyridazinone 1 (Pyr1), is a potent TRPC4 and TRPC4/C5 inhibitor [236,237]. Pyr3 and Pyr10 are known to inhibit TRPC3 [230]. Another sub micromolar potent inhibitor of TRPC3 and -C6 is GSK2833503, which is known to be more than 100-fold more selective against TRPC3 and TRPC6 than other Ca^2+^ channels [240,241]. The BI 749327 inhibitor was developed to specifically inhibit TRPC6. It is an orally bioavailable inhibitor that does not inhibit TRPC5 or other TRP channels [242]. The (+)-larixol derivative SH045 is also used to inhibit TRPC6, with good selectivity [243]. TRPC6 can also be inhibited by a nanomolar inhibitor and orally bioavailable SAR7334 [238] or by its analogue AM-1473 [244].

Some studies are ongoing, such as the TRPC5 channel inhibitor GFB887, tested in Phase I on healthy volunteers, and now in Phase II for patients with diabetic nephropathy or with focal segmental glomerulosclerosis [250]. However, research on TRPC inhibitors is at an early stage but should be accelerated by recent cryo-EM structure discovery.

As described in this part, the selectivity of these molecules is not enough to discriminate each of the TRPC isoforms. In vitro, these inhibitors may be used at specific concentrations to ensure selectivity, but in vivo the use of these molecules is challenging due to their lack of selectivity. Additionally, the mechanism of action or channel specificity remains unclear for some of these pharmacological agents. Moreover, some compounds are not stable in vivo, which prevents their use in preclinical or clinical research. Progress in the identification of SOCs involved in the pathogenesis of PAH is essential to develop specific therapeutic tools. The recent advance in the determination of cryo-EM structures of TRPC channels with sufficient resolution to allow the identification of specific bounding site of small molecules [251,252] should help to develop more potent selective inhibitors or activators, since the need of selective molecules is crucial for the development of innovative therapy in various diseases, including PAH. Despite important progress in the selectivity of the molecules described in this review, the development of more selective pharmacological tools to selectively inhibit each SOC isoform could facilitate the emergence of innovative therapies concerning multiple diseases, including PAH.

## 4. Conclusions

PAH represents a major human and social burden because this orphan disease is associated with a poor prognosis, affects children and young adults, and has no cure treatment except lung transplantation for eligible patients. The data presented in the present review support evidence of the involvement of SOCs in the pathogenesis of PAH, and each of these channels could be considered a potential new therapeutic target for PAH. Nevertheless, these channels are ubiquitous. Consequently, specific pharmacological tools towards a target organ should be carefully considered in preclinical experiments to identify eventual side effects.

## Figures and Tables

**Table 1:** TRPC channels properties.

Channel	Selectivity P_Ca_/P_Na_	Conductance (pS)	Activation Mechanism	References
**TRPC1**	1	16	Store depletion, GPCR–PLC pathway, membrane stretching,	[60,95,96]
**TRPC2**	2.7	42	DAG	[97]
**TRPC3**	1.6	66	DAG, store depletion, PKC phosphorylation, membrane stretching	[73,94,98,99,100]
**TRPC4**	1.1–7.7	42	Store depletion, GPCR–PLC pathway	[101,102,103]
**TRPC5**	1.8–9.5	63	Store depletion, GPCR–PLC pathway	[101,103,104]
**TRPC6**	5	35	DAG, membrane stretching	[77,97,105]
**TRPC7**	1.9	25–50	DAG, store depletion	[50,94,106]

DAG, diacylglycerol; GPCR, G-protein coupled receptor; P_Ca_/P_Na_, permeability ratio between Ca^2+^ and Na^+^; PKC, protein kinase C; PLC, phospholipase C.

**Table 2:** TRPC channels expression in PASMC.

	TRPC1		TRPC3		TRPC4		TRPC5		TRPC6		References
Expression	mRNA	Protein	mRNA	Protein	mRNA	Protein	mRNA	Protein	mRNA	Protein	
**Human**	✓	✓	✓	✓	✓	✓	-	-	✓	✓	[141,142,143,144,145,146]
**Rat**	✓	✓	✓	✓	✓	✓	✓	-	✓	✓	[147,148,149,150]
**Mouse**	✓	✓	✓	-	✓	✓	-	-	✓	✓	[133,151,152,153,154,155,156]

-, no data available.

**Table 3:** Changes in SOC expression (mRNA and protein) and function in PASMC from PAH and from experimental models of PH.

	mRNA	Protein	SOCE	hPASMC	Rodents Models	Consequences	References
STIM1	-	↑	-	Hypoxia		Increased proliferation of rat PASMC Increased NFATc3 nuclear translocation	[140,173]
	↑	↑	-		Rat CH Distal PA		
STIM2	-	↑	↑	PAH		Increased proliferation of iPAH hPASMC	[136,173]
	-	↑	-	Hypoxia			
Orai1	-	↑	-	Hypoxia			[134,173]
	↑	↑	↑		Rat CH PA Rat CH PASMC		
Orai2	-	↑	-	Hypoxia			[134,173]
	↑	↑	↑		Rat CH PA Rat CH PASMC		
TRPC1	↑	↑	↑		Rat CH/MCT PA Rat CH PASMC	Reduced CH-induced PH phenotype and PASMC proliferation in *trpc1^−/−^* mice Pharmacological inhibition normalized vascular tone in PAs of CH-induced PH rats Increased murine PASMC proliferation	[145,152,153,185,186]
	↑	-	-		Mouse PASMC exposed to hypoxia		
TRPC3	↑	↑	↑	PAH			[173]
TRPC4	↑	↑	↑		Rat SU/Hx PA Rat SU/Hx PASMC	*trpc4* gene deletion reduces PH	[186,187]
TRPC6	↑	↑	-	PAH		*hPASMC* proliferation *trpc6* gene deletion reduces CH-induced PH in mice Pharmacological inhibition normalized vascular tone in PAs of CH-induced PH rats	[145,146,152,173]
	-	↑	-	Hypoxia			
	↑	↑	↑		Rat CH PA and PASMC		
SOCE	-	-	↑	PAH		Increased STIM1–Orai1–TRPC1 interaction in hypoxic mouse PASMC	[151,157,172]
	-	-	↑		Mouse PASMC exposed to hypoxia		

The upwards pointing arrow shows an increase; -, no available information.

**Table 4:** SOCs inhibitors used as research tool.

Compound	Mode of Action	IC50	Side Effects	References
Orai1 Inhibitor				
YM-58483 (BTP2 or Pyr2)	-	10–590 nM	Inhibits TRPC3 and -C6 (IC50: 0.3 µM) Activates TRPM4 channels (EC50: 8 nM) Inhibit Orai2 and Orai3 at 10 µM	[214,215,216,217,218,219]
GSK7975A and GSK-5503A	Potentially allosteric effect on the selectivity filter of Orai	4 µM	Orai2 and Orai3 at 10 µM, L-type Ca^2+^ (IC50: 8 µM), and TRPV6 channels	[219,220]
Synta-66	Binds TM1 and TM3 helices and the extracellular loop segments	26 nM-3 µM	Potentiate Orai2 at 10 µM	[219,221,222,223,224]
JPIII	-	244 nM	-	[225]
Gd^3+^ or La^3+^	Binds extracellular loop of Orai1	200 nM	Inhibit Orai2/3	[219,226]
AnCoA4	Binds the C-terminus of Orai1	880 nM	-	[227]
5J-4	-	10 µM	-	[228]
**STIM1 Inhibitor**				
ML-9	Inhibit STIM1 puncta formation	10 µM	Inhibit Myosin light chain kinase	[229]
**TRPC3 Inhibitor**				
Pyr3	Direct binding	0.7 μM	-	[230]
Pyr10	-	0.72 µM	-	[218]
**TRPC4 Inhibitor**				
ML-204	-	1–3 μM	Inhibit TRPC5 and weakly TRPC6	[231]
HC-070	Direct binding	9.3 nM	Inhibit TRPC4 (IC50:46 nM) and TRPC3 (IC50: 1 µM)	[232,233]
HC-608 (Pico145)	-	0.35 nM	Inhibit TRPC5 (IC50: 1.3 nM), TRPC1-4 complex (IC50: 0.03 nM) and TRPC1-5 complex (IC50: 0.2 nM)	[234]
**TRPC5 Inhibitor**				
AC1903	-	13.6 μM	Inhibit TRPC4 (IC50 > 100 µM)	[235]
GFB-8438	Direct binding	0.18 µM	Inhibit TRPC4 (IC50: 0.29 µM)	[236,237]
**TRPC6 Inhibitor**				
SAR7334	-	9.5 nM	Inhibit TRPC3 (IC50: 282 nM) and TRPC7 (IC50: 226 nM)	[238]
SKF-96365	-	10 µM	-	[239]
GSK2833503	-	3 nM	Inhibit TRPC3 (IC50: 21 nM)	[240,241]
BI 749327	-	19 nM	-	[242]
SH045	-	7.9 nM	Inhibit TRPC3 (IC50: 282 nM) and TRPC7 (IC50: 226 nM)	[243]
AM-1473	Direct binding	0.22 nM	-	[244]

Inhibitors are classified according to their smallest IC50. EC50, half maximal effective concentration; IC50, half maximal inhibitory concentration; -, no data available.

## Data Availability

Not applicable.

## References

[1] Bootman M.D., Collins T.J., Peppiatt C.M., Prothero L.S., MacKenzie L., De Smet P., Travers M., Tovey S.C., Seo J.T., Berridge M.J. (2001). Calcium Signalling—an Overview. Semin. Cell Dev. Biol..

[2] Berridge M.J., Bootman M.D., Roderick H.L. (2003). Calcium Signalling: Dynamics, Homeostasis and Remodelling. Nat. Rev. Mol. Cell Biol..

[3] Clapham D.E. (2007). Calcium Signaling. Cell.

[4] Putney J.W. (1986). A Model for Receptor-Regulated Calcium Entry. Cell Calcium.

[5] Hoth M., Penner R. (1992). Depletion of Intracellular Calcium Stores Activates a Calcium Current in Mast Cells. Nature.

[6] Zweifach A., Lewis R.S. (1995). Slow Calcium-Dependent Inactivation of Depletion-Activated Calcium Current. Store-Dependent and -Independent Mechanisms. J. Biol. Chem..

[7] Parekh A.B., Penner R. (1997). Store Depletion and Calcium Influx. Physiol. Rev..

[8] Roos J., DiGregorio P.J., Yeromin A.V., Ohlsen K., Lioudyno M., Zhang S., Safrina O., Kozak J.A., Wagner S.L., Cahalan M.D. (2005). STIM1, an Essential and Conserved Component of Store-Operated Ca^2+^ Channel Function. J. Cell Biol..

[9] Liou J., Kim M.L., Heo W.D., Jones J.T., Myers J.W., Ferrell J.E., Meyer T. (2005). STIM Is a Ca^2+^ Sensor Essential for Ca^2+^-Store-Depletion-Triggered Ca^2+^ Influx. Curr. Biol..

[10] Feske S., Gwack Y., Prakriya M., Srikanth S., Puppel S.-H., Tanasa B., Hogan P.G., Lewis R.S., Daly M., Rao A. (2006). A Mutation in Orai1 Causes Immune Deficiency by Abrogating CRAC Channel Function. Nature.

[11] Williams R.T., Manji S.S., Parker N.J., Hancock M.S., Van Stekelenburg L., Eid J.P., Senior P.V., Kazenwadel J.S., Shandala T., Saint R. (2001). Identification and Characterization of the STIM (Stromal Interaction Molecule) Gene Family: Coding for a Novel Class of Transmembrane Proteins. Biochem. J..

[12] Stathopulos P.B., Li G.-Y., Plevin M.J., Ames J.B., Ikura M. (2006). Stored Ca^2+^ Depletion-Induced Oligomerization of Stromal Interaction Molecule 1 (STIM1) via the EF-SAM Region. J. Biol. Chem..

[13] Spassova M.A., Soboloff J., He L.-P., Xu W., Dziadek M.A., Gill D.L. (2006). STIM1 Has a Plasma Membrane Role in the Activation of Store-Operated Ca^2+^ Channels. Proc. Natl. Acad. Sci. USA.

[14] Mercer J.C., DeHaven W.I., Smyth J.T., Wedel B., Boyles R.R., Bird G.S., Putney J.W. (2006). Large store-operated calcium-selective currents due to co-expression of orai1 or orai2 with the intracellular calcium sensor, stim1. J. Biol. Chem..

[15] Stathopulos P.B., Zheng L., Ikura M. (2009). Stromal Interaction Molecule (STIM) 1 and STIM2 Calcium Sensing Regions Exhibit Distinct Unfolding and Oligomerization Kinetics. J. Biol. Chem..

[16] Kim C.A., Bowie J.U. (2003). SAM Domains: Uniform Structure, Diversity of Function. Trends Biochem. Sci..

[17] Ma G., Wei M., He L., Liu C., Wu B., Zhang S.L., Jing J., Liang X., Senes A., Tan P. (2015). Inside-out Ca^2+^ Signalling Prompted by STIM1 Conformational Switch. Nat. Commun..

[18] Stathopulos P.B., Schindl R., Fahrner M., Zheng L., Gasmi-Seabrook G.M., Muik M., Romanin C., Ikura M. (2013). STIM1/Orai1 Coiled-Coil Interplay in the Regulation of Store-Operated Calcium Entry. Nat. Commun..

[19] Fahrner M., Muik M., Schindl R., Butorac C., Stathopulos P., Zheng L., Jardin I., Ikura M., Romanin C. (2014). A Coiled-Coil Clamp Controls Both Conformation and Clustering of Stromal Interaction Molecule 1 (STIM1). J. Biol. Chem..

[20] Muik M., Fahrner M., Schindl R., Stathopulos P., Frischauf I., Derler I., Plenk P., Lackner B., Groschner K., Ikura M. (2011). STIM1 Couples to ORAI1 via an Intramolecular Transition into an Extended Conformation. EMBO J..

[21] Kim M.S., Zeng W., Yuan J.P., Shin D.M., Worley P.F., Muallem S. (2009). Native Store-Operated Ca^2+^ Influx Requires the Channel Function of Orai1 and TRPC1. J. Biol. Chem..

[22] Yuan J.P., Zeng W., Dorwart M.R., Choi Y.-J., Worley P.F., Muallem S. (2009). SOAR and the Polybasic STIM1 Domains Gate and Regulate Orai Channels. Nat. Cell Biol..

[23] Park C.Y., Shcheglovitov A., Dolmetsch R. (2010). The CRAC Channel Activator STIM1 Binds and Inhibits L-Type Voltage-Gated Calcium Channels. Science.

[24] Frischauf I., Muik M., Derler I., Bergsmann J., Fahrner M., Schindl R., Groschner K., Romanin C. (2009). Molecular Determinants of the Coupling between STIM1 and Orai Channels: Differential Activation Of Orai1–3 Channels by a Stim1 Coiled-Coil Mutant. J. Biol. Chem..

[25] Zeng W., Yuan J.P., Kim M.S., Choi Y.J., Huang G.N., Worley P.F., Muallem S. (2008). STIM1 Gates TRPC Channels but Not Orai1 by Electrostatic Interaction. Mol. Cell.

[26] Liou J., Fivaz M., Inoue T., Meyer T. (2007). Live-Cell Imaging Reveals Sequential Oligomerization and Local Plasma Membrane Targeting of Stromal Interaction Molecule 1 after Ca^2+^ Store Depletion. Proc. Natl. Acad. Sci. USA.

[27] Park C.Y., Hoover P.J., Mullins F.M., Bachhawat P., Covington E.D., Raunser S., Walz T., Garcia K.C., Dolmetsch R.E., Lewis R.S. (2009). STIM1 Clusters and Activates CRAC Channels via Direct Binding of a Cytosolic Domain to Orai1. Cell.

[28] Brandman O., Liou J., Park W.S., Meyer T. (2007). STIM2 Is a Feedback Regulator That Stabilizes Basal Cytosolic and Endoplasmic Reticulum Ca^2+^ Levels. Cell.

[29] Zhou Y., Mancarella S., Wang Y., Yue C., Ritchie M., Gill D.L., Soboloff J. (2009). The Short N-Terminal Domains of STIM1 and STIM2 Control the Activation Kinetics of Orai1 Channels. J. Biol. Chem..

[30] Darbellay B., Arnaudeau S., Bader C.R., Konig S., Bernheim L. (2011). STIM1L Is a New Actin-Binding Splice Variant Involved in Fast Repetitive Ca^2+^ Release. J. Cell Biol..

[31] Ramesh G., Jarzembowski L., Schwarz Y., Poth V., Konrad M., Knapp M.L., Schwär G., Lauer A.A., Grimm M.O.W., Alansary D. (2021). A Short Isoform of STIM1 Confers Frequency-Dependent Synaptic Enhancement. Cell Rep..

[32] Miederer A.-M., Alansary D., Schwär G., Lee P.-H., Jung M., Helms V., Niemeyer B.A. (2015). A STIM2 Splice Variant Negatively Regulates Store-Operated Calcium Entry. Nat. Commun..

[33] Rana A., Yen M., Sadaghiani A.M., Malmersjö S., Park C.Y., Dolmetsch R.E., Lewis R.S. (2015). Alternative Splicing Converts STIM2 from an Activator to an Inhibitor of Store-Operated Calcium Channels. J. Cell Biol..

[34] Feske S., Prakriya M., Rao A., Lewis R.S. (2005). A Severe Defect in CRAC Ca^2+^ Channel Activation and Altered K+ Channel Gating in T Cells from Immunodeficient Patients. J. Exp. Med..

[35] Zhang S.L., Yeromin A.V., Zhang X.H.-F., Yu Y., Safrina O., Penna A., Roos J., Stauderman K.A., Cahalan M.D. (2006). Genome-Wide RNAi Screen of Ca^2+^ Influx Identifies Genes That Regulate Ca^2+^ Release-Activated Ca^2+^ Channel Activity. Proc. Natl. Acad. Sci. USA.

[36] Yeromin A.V., Zhang S.L., Jiang W., Yu Y., Safrina O., Cahalan M.D. (2006). Molecular Identification of the CRAC Channel by Altered Ion Selectivity in a Mutant of Orai. Nature.

[37] Vig M., Peinelt C., Beck A., Koomoa D.L., Rabah D., Koblan-Huberson M., Kraft S., Turner H., Fleig A., Penner R. (2006). CRACM1 Is a Plasma Membrane Protein Essential for Store-Operated Ca^2+^ Entry. Science.

[38] Prakriya M., Feske S., Gwack Y., Srikanth S., Rao A., Hogan P.G. (2006). Orai1 Is an Essential Pore Subunit of the CRAC Channel. Nature.

[39] Hou X., Pedi L., Diver M.M., Long S.B. (2012). Crystal Structure of the Calcium Release-Activated Calcium Channel Orai. Science.

[40] Derler I., Plenk P., Fahrner M., Muik M., Jardin I., Schindl R., Gruber H.J., Groschner K., Romanin C. (2013). The Extended Transmembrane Orai1 N-Terminal (ETON) Region Combines Binding Interface and Gate for Orai1 Activation by STIM1. J. Biol. Chem..

[41] Gwack Y., Srikanth S., Feske S., Cruz-Guilloty F., Oh-hora M., Neems D.S., Hogan P.G., Rao A. (2007). Biochemical and Functional Characterization of Orai Proteins. J. Biol. Chem..

[42] Gross S.A., Wissenbach U., Philipp S.E., Freichel M., Cavalié A., Flockerzi V. (2007). Murine ORAI2 Splice Variants Form Functional Ca^2+^ Release-Activated Ca^2+^ (CRAC) Channels. J. Biol. Chem..

[43] Motiani R.K., Abdullaev I.F., Trebak M. (2010). A Novel Native Store-Operated Calcium Channel Encoded by Orai3: Selective requirement of orai3 versus orai1 in estrogen receptor-positive versus estrogen receptor-negative breast cancer cells. J. Biol. Chem..

[44] Lis A., Peinelt C., Beck A., Parvez S., Monteilh-Zoller M., Fleig A., Penner R. (2007). CRACM1, CRACM2, and CRACM3 Are Store-Operated Ca^2+^ Channels with Distinct Functional Properties. Curr. Biol..

[45] Hoth M., Niemeyer B.A., Prakriya M. (2013). Chapter Ten—The Neglected CRAC Proteins: Orai2, Orai3, and STIM2. Current Topics in Membranes.

[46] Fukushima M., Tomita T., Janoshazi A., Putney J.W. (2012). Alternative Translation Initiation Gives Rise to Two Isoforms of Orai1 with Distinct Plasma Membrane Mobilities. J. Cell Sci..

[47] Desai P.N., Zhang X., Wu S., Janoshazi A., Bolimuntha S., Putney J.W., Trebak M. (2015). Multiple Types of Calcium Channels Arising from Alternative Translation Initiation of the Orai1 Message. Sci. Signal..

[48] Montell C. (2011). The History of TRP Channels, a Commentary and Reflection. Pflug. Arch.—Eur. J. Physiol..

[49] Li H., Wang Y. (2017). TRP Channel Classification. Transient Receptor Potential Canonical Channels and Brain Diseases.

[50] Owsianik G., Talavera K., Voets T., Nilius B. (2006). Permeation and Selectivity of TRP Channels. Annu. Rev. Physiol..

[51] Vazquez G., Wedel B.J., Aziz O., Trebak M., Putney J.W. (2004). The Mammalian TRPC Cation Channels. Biochim. Et Biophys. Acta (BBA)—Mol. Cell Res..

[52] Salido G.M., Sage S.O., Rosado J.A. (2009). TRPC Channels and Store-Operated Ca^2+^ Entry. Biochim. Et Biophys. Acta (BBA)—Mol. Cell Res..

[53] Tang Q., Guo W., Zheng L., Wu J.-X., Liu M., Zhou X., Zhang X., Chen L. (2018). Structure of the Receptor-Activated Human TRPC6 and TRPC3 Ion Channels. Cell Res..

[54] Wang H., Cheng X., Tian J., Xiao Y., Tian T., Xu F., Hong X., Zhu M.X. (2020). TRPC Channels: Structure, Function, Regulation and Recent Advances in Small Molecular Probes. Pharmacol. Ther..

[55] Lepage P.K., Lussier M.P., Barajas-Martinez H., Bousquet S.M., Blanchard A.P., Francoeur N., Dumaine R., Boulay G. (2006). Identification of Two Domains Involved in the Assembly of Transient Receptor Potential Canonical Channels. J. Biol. Chem..

[56] Gaudet R. (2008). A Primer on Ankyrin Repeat Function in TRP Channels and Beyond. Mol. Biosyst..

[57] Boulay G., Brown D.M., Qin N., Jiang M., Dietrich A., Zhu M.X., Chen Z., Birnbaumer M., Mikoshiba K., Birnbaumer L. (1999). Modulation of Ca^2+^ Entry by Polypeptides of the Inositol 1,4,5-Trisphosphate Receptor (IP3R) That Bind Transient Receptor Potential (TRP): Evidence for Roles of TRP and IP3R in Store Depletion-Activated Ca^2+^ Entry. Proc. Natl. Acad. Sci. USA.

[58] Zhang Z., Tang J., Tikunova S., Johnson J.D., Chen Z., Qin N., Dietrich A., Stefani E., Birnbaumer L., Zhu M.X. (2001). Activation of Trp3 by Inositol 1,4,5-Trisphosphate Receptors through Displacement of Inhibitory Calmodulin from a Common Binding Domain. Proc. Natl. Acad. Sci. USA.

[59] Wedel B.J., Vazquez G., McKay R.R., Bird G.S.T., Putney J.W. (2003). A Calmodulin/Inositol 1,4,5-Trisphosphate (IP3) Receptor-Binding Region Targets TRPC3 to the Plasma Membrane in a Calmodulin/IP3 Receptor-Independent Process. J. Biol. Chem..

[60] Zitt C., Zobel A., Obukhov A.G., Harteneck C., Kalkbrenner F., Lückhoff A., Schultz G. (1996). Cloning and Functional Expression of a Human Ca^2+^-Permeable Cation Channel Activated by Calcium Store Depletion. Neuron.

[61] Zhu X., Jiang M., Peyton M., Boulay G., Hurst R., Stefani E., Birnbaumer L. (1996). Trp, a Novel Mammalian Gene Family Essential for Agonist-Activated Capacitative Ca^2+^ Entry. Cell.

[62] Liu X., Wang W., Singh B.B., Lockwich T., Jadlowiec J., O’Connell B., Wellner R., Zhu M.X., Ambudkar I.S. (2000). Trp1, a Candidate Protein for the Store-Operated Ca^2+^ Influx Mechanism in Salivary Gland Cells. J. Biol. Chem..

[63] Mehta D., Ahmmed G.U., Paria B.C., Holinstat M., Voyno-Yasenetskaya T., Tiruppathi C., Minshall R.D., Malik A.B. (2003). RhoA Interaction with Inositol 1,4,5-Trisphosphate Receptor and Transient Receptor Potential Channel-1 Regulates Ca^2+^ Entry: Role in signaling increased endothelial permeability. J. Biol. Chem..

[64] Antigny F., Koenig S., Bernheim L., Frieden M. (2013). During Post-Natal Human Myogenesis, Normal Myotube Size Requires TRPC1- and TRPC4-Mediated Ca^2+^ Entry. J. Cell Sci..

[65] Sabourin J., Lamiche C., Vandebrouck A., Magaud C., Rivet J., Cognard C., Bourmeyster N., Constantin B. (2009). Regulation of TRPC1 and TRPC4 Cation Channels Requires an Alpha1-Syntrophin-Dependent Complex in Skeletal Mouse Myotubes. J. Biol. Chem..

[66] Antigny F., Sabourin J., Saüc S., Bernheim L., Koenig S., Frieden M. (2017). TRPC1 and TRPC4 Channels Functionally Interact with STIM1L to Promote Myogenesis and Maintain Fast Repetitive Ca^2+^ Release in Human Myotubes. Biochim. Biophys. Acta Mol. Cell Res..

[67] Brownlow S.L., Harper A.G.S., Harper M.T., Sage S.O. (2004). A Role for HTRPC1 and Lipid Raft Domains in Store-Mediated Calcium Entry in Human Platelets. Cell Calcium.

[68] Liu X., Cheng K.T., Bandyopadhyay B.C., Pani B., Dietrich A., Paria B.C., Swaim W.D., Beech D., Yildrim E., Singh B.B. (2007). Attenuation of Store-Operated Ca^2+^ Current Impairs Salivary Gland Fluid Secretion in TRPC1(−/−) Mice. Proc. Natl. Acad. Sci. USA.

[69] Hong J.H., Li Q., Kim M.S., Shin D.M., Feske S., Birnbaumer L., Cheng K.T., Ambudkar I.S., Muallem S. (2011). Polarized but Differential Localization and Recruitment of STIM1/Orai1 and STIM1/TRPC Channels in Secretory Cells. Traffic.

[70] Ma X., Cheng K.-T., Wong C.-O., O’Neil R.G., Birnbaumer L., Ambudkar I.S., Yao X. (2011). Heteromeric TRPV4-C1 Channels Contribute to Store-Operated Ca^2+^ Entry in Vascular Endothelial Cells. Cell Calcium.

[71] Vazquez G., Wedel B.J., Trebak M., Bird G.S.J., Putney J.W. (2003). Expression Level of the Canonical Transient Receptor Potential 3 (TRPC3) Channel Determines Its Mechanism of Activation. J. Biol. Chem..

[72] Wu X., Zagranichnaya T.K., Gurda G.T., Eves E.M., Villereal M.L. (2004). A TRPC1/TRPC3-Mediated Increase in Store-Operated Calcium Entry Is Required for Differentiation of H19-7 Hippocampal Neuronal Cells. J. Biol. Chem..

[73] Antigny F., Jousset H., König S., Frieden M. (2011). Thapsigargin Activates Ca^2+^ Entry Both by Store-Dependent, STIM1/Orai1-Mediated, and Store-Independent, TRPC3/PLC/PKC-Mediated Pathways in Human Endothelial Cells. Cell Calcium.

[74] Yuan J.P., Zeng W., Huang G.N., Worley P.F., Muallem S. (2007). STIM1 Heteromultimerizes TRPC Channels to Determine Their Function as Store-Operated Channels. Nat. Cell Biol..

[75] Kim M.S., Hong J.H., Li Q., Shin D.M., Abramowitz J., Birnbaumer L., Muallem S. (2009). Deletion of TRPC3 in Mice Reduces Store-Operated Ca^2+^ Influx and the Severity of Acute Pancreatitis. Gastroenterology.

[76] Kim M.S., Lee K.P., Yang D., Shin D.M., Abramowitz J., Kiyonaka S., Birnbaumer L., Mori Y., Muallem S. (2011). Genetic and Pharmacological Inhibition of the Ca^2+^ Influx Channel TRPC3 Protects Secretory Epithelia from Ca^2+^-Dependent Toxicity. Gastroenterology.

[77] Kinoshita M., Akaike A., Satoh M., Kaneko S. (2000). Positive Regulation of Capacitative Ca^2+^ Entry by Intracellular Ca^2+^ in Xenopus Oocytes Expressing Rat TRP4. Cell Calcium.

[78] Philipp S., Trost C., Warnat J., Rautmann J., Himmerkus N., Schroth G., Kretz O., Nastainczyk W., Cavalie A., Hoth M. (2000). TRP4 (CCE1) Protein Is Part of Native Calcium Release-Activated Ca^2+^-like Channels in Adrenal Cells. J. Biol. Chem..

[79] Yang H., Mergler S., Sun X., Wang Z., Lu L., Bonanno J.A., Pleyer U., Reinach P.S. (2005). TRPC4 Knockdown Suppresses Epidermal Growth Factor-Induced Store-Operated Channel Activation and Growth in Human Corneal Epithelial Cells. J. Biol. Chem..

[80] Fatherazi S., Presland R.B., Belton C.M., Goodwin P., Al-Qutub M., Trbic Z., Macdonald G., Schubert M.M., Izutsu K.T. (2007). Evidence That TRPC4 Supports the Calcium Selective I(CRAC)-like Current in Human Gingival Keratinocytes. Pflug. Arch..

[81] Sabourin J., Bartoli F., Antigny F., Gomez A.M., Benitah J.-P. (2016). Transient Receptor Potential Canonical (TRPC)/Orai1-Dependent Store-Operated Ca^2+^ Channels: New targets of aldosterone in cardiomyocytes. J. Biol. Chem..

[82] Sundivakkam P.C., Freichel M., Singh V., Yuan J.P., Vogel S.M., Flockerzi V., Malik A.B., Tiruppathi C. (2012). The Ca^2+^ Sensor Stromal Interaction Molecule 1 (STIM1) Is Necessary and Sufficient for the Store-Operated Ca^2+^ Entry Function of Transient Receptor Potential Canonical (TRPC) 1 and 4 Channels in Endothelial Cells. Mol. Pharmacol..

[83] Freichel M., Suh S.H., Pfeifer A., Schweig U., Trost C., Weißgerber P., Biel M., Philipp S., Freise D., Droogmans G. (2001). Lack of an Endothelial Store-Operated Ca^2+^ Current Impairs Agonist-Dependent Vasorelaxation in TRP4^−/−^ Mice. Nat. Cell Biol..

[84] Tiruppathi C., Freichel M., Vogel S.M., Paria B.C., Mehta D., Flockerzi V., Malik A.B. (2002). Impairment of Store-Operated Ca^2+^ Entry in TRPC4−/− Mice Interferes With Increase in Lung Microvascular Permeability. Circ. Res..

[85] Xu S.-Z., Boulay G., Flemming R., Beech D.J. (2006). E3-Targeted Anti-TRPC5 Antibody Inhibits Store-Operated Calcium Entry in Freshly Isolated Pial Arterioles. Am. J. Physiol.-Heart Circ. Physiol..

[86] Domínguez-Rodríguez A., Mayoral-Gonzalez I., Avila-Medina J., de Rojas-de Pedro E.S., Calderón-Sánchez E., Díaz I., Hmadcha A., Castellano A., Rosado J.A., Benitah J.-P. (2018). Urocortin-2 Prevents Dysregulation of Ca^2+^ Homeostasis and Improves Early Cardiac Remodeling After Ischemia and Reperfusion. Front. Physiol..

[87] Bréchard S., Melchior C., Plançon S., Schenten V., Tschirhart E.J. (2008). Store-Operated Ca^2+^ Channels Formed by TRPC1, TRPC6 and Orai1 and Non-Store-Operated Channels Formed by TRPC3 Are Involved in the Regulation of NADPH Oxidase in HL-60 Granulocytes. Cell Calcium.

[88] Jardín I., Redondo P.C., Salido G.M., Rosado J.A. (2008). Phosphatidylinositol 4,5-Bisphosphate Enhances Store-Operated Calcium Entry through HTRPC6 Channel in Human Platelets. Biochim. Biophys. Acta.

[89] Jardin I., Gómez L.J., Salido G.M., Rosado J.A. (2009). Dynamic Interaction of HTRPC6 with the Orai1-STIM1 Complex or HTRPC3 Mediates Its Role in Capacitative or Non-Capacitative Ca^2+^ Entry Pathways. Biochem. J..

[90] Berna-Erro A., Galan C., Dionisio N., Gomez L.J., Salido G.M., Rosado J.A. (2012). Capacitative and Non-Capacitative Signaling Complexes in Human Platelets. Biochim. Et Biophys. Acta (BBA)—Mol. Cell Res..

[91] El Boustany C., Bidaux G., Enfissi A., Delcourt P., Prevarskaya N., Capiod T. (2008). Capacitative Calcium Entry and Transient Receptor Potential Canonical 6 Expression Control Human Hepatoma Cell Proliferation. Hepatology.

[92] DeHaven W.I., Jones B.F., Petranka J.G., Smyth J.T., Tomita T., Bird G.S., Putney J.W. (2009). TRPC Channels Function Independently of STIM1 and Orai1. J. Physiol..

[93] Zhang X., Trebak M. (2014). Transient Receptor Potential Canonical 7 (TRPC7): A Diacylglycerol-Activated Non-Selective Cation Channel. Handb. Exp. Pharmacol..

[94] Lemonnier L., Trebak M., Putney J.W. (2008). Complex Regulation of the TRPC3,6,7 Channel Subfamily by Diacylglycerol and Phosphatidylinositol 4,5-Bisphosphate. Cell Calcium.

[95] Maroto R., Raso A., Wood T.G., Kurosky A., Martinac B., Hamill O.P. (2005). TRPC1 Forms the Stretch-Activated Cation Channel in Vertebrate Cells. Nat. Cell. Biol..

[96] Wes P.D., Chevesich J., Jeromin A., Rosenberg C., Stetten G., Montell C. (1995). TRPC1, a Human Homolog of a Drosophila Store-Operated Channel. Proc. Natl. Acad. Sci. USA.

[97] Lucas P., Ukhanov K., Leinders-Zufall T., Zufall F. (2003). A Diacylglycerol-Gated Cation Channel in Vomeronasal Neuron Dendrites Is Impaired in TRPC2 Mutant Mice: Mechanism of Pheromone Transduction. Neuron.

[98] Zitt C., Obukhov A.G., Strübing C., Zobel A., Kalkbrenner F., Lückhoff A., Schultz G. (1997). Expression of TRPC3 in Chinese Hamster Ovary Cells Results in Calcium-Activated Cation Currents Not Related to Store Depletion. J. Cell Biol..

[99] Hofmann T., Obukhov A.G., Schaefer M., Harteneck C., Gudermann T., Schultz G. (1999). Direct Activation of Human TRPC6 and TRPC3 Channels by Diacylglycerol. Nature.

[100] Kamouchi M., Philipp S., Flockerzi V., Wissenbach U., Mamin A., Raeymaekers L., Eggermont J., Droogmans G., Nilius B. (1999). Properties of Heterologously Expressed HTRP3 Channels in Bovine Pulmonary Artery Endothelial Cells. J. Physiol..

[101] Schaefer M., Plant T.D., Obukhov A.G., Hofmann T., Gudermann T., Schultz G. (2000). Receptor-Mediated Regulation of the Nonselective Cation Channels TRPC4 and TRPC5. J. Biol. Chem..

[102] Kew J.N.C., Davies C.H. (2010). Ion Channels: From Structure to Function.

[103] Feng S. (2017). TRPC Channel Structure and Properties. Adv. Exp. Med. Biol..

[104] Jung S., Mühle A., Schaefer M., Strotmann R., Schultz G., Plant T.D. (2003). Lanthanides Potentiate TRPC5 Currents by an Action at Extracellular Sites Close to the Pore Mouth. J. Biol. Chem..

[105] Spassova M.A., Hewavitharana T., Xu W., Soboloff J., Gill D.L. (2006). A Common Mechanism Underlies Stretch Activation and Receptor Activation of TRPC6 Channels. Proc. Natl. Acad. Sci. USA.

[106] Okada T., Inoue R., Yamazaki K., Maeda A., Kurosaki T., Yamakuni T., Tanaka I., Shimizu S., Ikenaka K., Imoto K. (1999). Molecular and Functional Characterization of a Novel Mouse Transient Receptor Potential Protein Homologue TRP7. J. Biol. Chem..

[107] Berridge M.J., Irvine R.F. (1984). Inositol Trisphosphate, a Novel Second Messenger in Cellular Signal Transduction. Nature.

[108] Streb H., Irvine R.F., Berridge M.J., Schulz I. (1983). Release of Ca^2+^ from a Nonmitochondrial Intracellular Store in Pancreatic Acinar Cells by Inositol-1,4,5-Trisphosphate. Nature.

[109] Korzeniowski M.K., Manjarrés I.M., Varnai P., Balla T. (2010). Activation of STIM1-ORAI1 involves an intramolecular switching mechanism. Sci. Signal..

[110] Korzeniowski M.K., Wisniewski E., Baird B., Holowka D.A., Balla T. (2017). Molecular Anatomy of the Early Events in STIM1 Activation—Oligomerization or Conformational Change?. J. Cell Sci..

[111] Luik R.M., Wang B., Prakriya M., Wu M.M., Lewis R.S. (2008). Oligomerization of STIM1 Couples ER Calcium Depletion to CRAC Channel Activation. Nature.

[112] Hoover P.J., Lewis R.S. (2011). Stoichiometric Requirements for Trapping and Gating of Ca^2+^ Release-Activated Ca^2+^ (CRAC) Channels by Stromal Interaction Molecule 1 (STIM1). Proc. Natl. Acad. Sci. USA.

[113] Huang G.N., Zeng W., Kim J.Y., Yuan J.P., Han L., Muallem S., Worley P.F. (2006). STIM1 Carboxyl-Terminus Activates Native SOC, I Crac and TRPC1 Channels. Nat. Cell Biol..

[114] Lee K.P., Yuan J.P., So I., Worley P.F., Muallem S. (2010). STIM1-Dependent and STIM1-Independent Function of Transient Receptor Potential Canonical (TRPC) Channels Tunes Their Store-Operated Mode. J. Biol. Chem..

[115] Cheng K.T., Liu X., Ong H.L., Swaim W., Ambudkar I.S. (2011). Local Ca^2+^ Entry Via Orai1 Regulates Plasma Membrane Recruitment of TRPC1 and Controls Cytosolic Ca^2+^ Signals Required for Specific Cell Functions. PLoS Biol..

[116] de Souza L.B., Ong H.L., Liu X., Ambudkar I.S. (2015). Fast Endocytic Recycling Determines TRPC1-STIM1 Clustering in ER-PM Junctions and Plasma Membrane Function of the Channel. Biochim. Biophys. Acta.

[117] Mignen O., Thompson J.L., Shuttleworth T.J. (2003). Ca^2+^ Selectivity and Fatty Acid Specificity of the Noncapacitative, Arachidonate-Regulated Ca^2+^ (ARC) Channels. J. Biol. Chem..

[118] González-Cobos J.C., Zhang X., Zhang W., Ruhle B., Motiani R.K., Schindl R., Muik M., Spinelli A.M., Bisaillon J.M., Shinde A.V. (2013). Store-Independent Orai1/3 Channels Activated by Intracrine LeukotrieneC4: Role in Neointimal Hyperplasia. Circ. Res..

[119] Mignen O., Thompson J.L., Shuttleworth T.J. (2008). Both Orai1 and Orai3 Are Essential Components of the Arachidonate-Regulated Ca^2+^-Selective (ARC) Channels. J. Physiol..

[120] Zhang X., Zhang W., González-Cobos J.C., Jardin I., Romanin C., Matrougui K., Trebak M. (2014). Complex Role of STIM1 in the Activation of Store-Independent Orai1/3 Channels. J. Gen. Physiol..

[121] Mignen O., Thompson J.L., Shuttleworth T.J. (2007). STIM1 Regulates Ca^2+^ Entry via Arachidonate-Regulated Ca^2+^-Selective (ARC) Channels without Store Depletion or Translocation to the Plasma Membrane. J. Physiol..

[122] Thompson J.L., Shuttleworth T.J. (2013). Molecular Basis of Activation of the Arachidonate-Regulated Ca^2+^ (ARC) Channel, a Store-Independent Orai Channel, by Plasma Membrane STIM1. J. Physiol..

[123] Humbert M., Guignabert C., Bonnet S., Dorfmüller P., Klinger J.R., Nicolls M.R., Olschewski A.J., Pullamsetti S.S., Schermuly R.T., Stenmark K.R. (2019). Pathology and Pathobiology of Pulmonary Hypertension: State of the Art and Research Perspectives. Eur. Respir. J..

[124] Simonneau G., Montani D., Celermajer D.S., Denton C.P., Gatzoulis M.A., Krowka M., Williams P.G., Souza R. (2019). Haemodynamic Definitions and Updated Clinical Classification of Pulmonary Hypertension. Eur. Respir. J..

[125] Galiè N., Humbert M., Vachiery J.-L., Gibbs S., Lang I., Torbicki A., Simonneau G., Peacock A., Noordegraaf A.V., Beghetti M. (2015). 2015 ESC/ERS Guidelines for the Diagnosis and Treatment of Pulmonary Hypertension: The Joint Task Force for the Diagnosis and Treatment of Pulmonary Hypertension of the European Society of Cardiology (ESC) and the European Respiratory Society (ERS)Endorsed by: Association for European Paediatric and Congenital Cardiology (AEPC), International Society for Heart and Lung Transplantation (ISHLT). Eur. Respir. J..

[126] Lau E.M.T., Giannoulatou E., Celermajer D.S., Humbert M. (2017). Epidemiology and Treatment of Pulmonary Arterial Hypertension. Nat. Rev. Cardiol..

[127] Farber H.W., Miller D.P., Poms A.D., Badesch D.B., Frost A.E., Muros-Le Rouzic E., Romero A.J., Benton W.W., Elliott C.G., McGoon M.D. (2015). Five-Year Outcomes of Patients Enrolled in the REVEAL Registry. Chest.

[128] Sitbon O., Gomberg-Maitland M., Granton J., Lewis M.I., Mathai S.C., Rainisio M., Stockbridge N.L., Wilkins M.R., Zamanian R.T., Rubin L.J. (2019). Clinical Trial Design and New Therapies for Pulmonary Arterial Hypertension. Eur. Respir. J..

[129] Antigny F., Girardin N., Frieden M. (2012). Transient Receptor Potential Canonical Channels Are Required for in Vitro Endothelial Tube Formation. J. Biol. Chem..

[130] Antigny F., Mercier O., Humbert M., Sabourin J. (2020). Excitation-Contraction Coupling and Relaxation Alteration in Right Ventricular Remodelling Caused by Pulmonary Arterial Hypertension. Arch. Cardiovasc. Dis..

[131] Sabourin J., Boet A., Rucker-Martin C., Lambert M., Gomez A.-M., Benitah J.-P., Perros F., Humbert M., Antigny F. (2018). Ca^2+^ Handling Remodeling and STIM1L/Orai1/TRPC1/TRPC4 Upregulation in Monocrotaline-Induced Right Ventricular Hypertrophy. J. Mol. Cell. Cardiol..

[132] Sankhe S., Manousakidi S., Antigny F., Arthur Ataam J., Bentebbal S., Ruchon Y., Lecerf F., Sabourin J., Price L., Fadel E. (2017). T-Type Ca^2+^ Channels Elicit pro-Proliferative and Anti-Apoptotic Responses through Impaired PP2A/Akt1 Signaling in PASMCs from Patients with Pulmonary Arterial Hypertension. Biochim. Biophys. Acta (BBA)—Mol. Cell Res..

[133] Ng L.C., Ramduny D., Airey J.A., Singer C.A., Keller P.S., Shen X.-M., Tian H., Valencik M., Hume J.R. (2010). Orai1 Interacts with STIM1 and Mediates Capacitative Ca^2+^ Entry in Mouse Pulmonary Arterial Smooth Muscle Cells. Am. J. Physiol.-Cell Physiol..

[134] Wang J. (2017). Orai1, 2, 3 and STIM1 Promote Store-Operated Calcium Entry in Pulmonary Arterial Smooth Muscle Cells. Cell Death Discov..

[135] Fernandez R.A., Wan J., Song S., Smith K.A., Gu Y., Tauseef M., Tang H., Makino A., Mehta D., Yuan J.X.-J. (2015). Upregulated Expression of STIM2, TRPC6, and Orai2 Contributes to the Transition of Pulmonary Arterial Smooth Muscle Cells from a Contractile to Proliferative Phenotype. Am. J. Physiol.-Cell Physiol..

[136] Song M.Y., Makino A., Yuan J.X.-J. (2011). STIM2 Contributes to Enhanced Store-Operated Ca^2+^ Entry in Pulmonary Artery Smooth Muscle Cells from Patients with Idiopathic Pulmonary Arterial Hypertension. Pulm. Circ..

[137] Ogawa A., Firth A.L., Smith K.A., Maliakal M.V., Yuan J.X.-J. (2011). PDGF Enhances Store-Operated Ca^2+^ Entry by Upregulating STIM1/Orai1 via Activation of Akt/MTOR in Human Pulmonary Arterial Smooth Muscle Cells. Am. J. Physiol.-Cell Physiol..

[138] Chen T.-X., Xu X.-Y., Zhao Z., Zhao F.-Y., Gao Y.-M., Yan X.-H., Wan Y. (2017). Hydrogen Peroxide Is a Critical Regulator of the Hypoxia-Induced Alterations of Store-Operated Ca^2+^ Entry into Rat Pulmonary Arterial Smooth Muscle Cells. Am. J. Physiol.-Lung Cell. Mol. Physiol..

[139] Smith K.A., Voiriot G., Tang H., Fraidenburg D.R., Song S., Yamamura H., Yamamura A., Guo Q., Wan J., Pohl N.M. (2015). Notch Activation of Ca^2+^ Signaling in the Development of Hypoxic Pulmonary Vasoconstriction and Pulmonary Hypertension. Am. J. Respir. Cell. Mol. Biol..

[140] Hou X., Chen J., Luo Y., Liu F., Xu G., Gao Y. (2013). Silencing of STIM1 Attenuates Hypoxia-Induced PASMCs Proliferation via Inhibition of the SOC/Ca^2+^/NFAT Pathway. Respir. Res..

[141] Zhang S., Remillard C.V., Fantozzi I., Yuan J.X.-J. (2004). ATP-Induced Mitogenesis Is Mediated by Cyclic AMP Response Element-Binding Protein-Enhanced TRPC4 Expression and Activity in Human Pulmonary Artery Smooth Muscle Cells. Am. J. Physiol.-Cell Physiol..

[142] Kunichika N., Yu Y., Remillard C.V., Platoshyn O., Zhang S., Yuan J.X.-J. (2004). Overexpression of TRPC1 Enhances Pulmonary Vasoconstriction Induced by Capacitative Ca^2+^ Entry. Am. J. Physiol.-Lung Cell. Mol. Physiol..

[143] Golovina V.A., Platoshyn O., Bailey C.L., Wang J., Limsuwan A., Sweeney M., Rubin L.J., Yuan J.X.-J. (2001). Upregulated TRP and Enhanced Capacitative Ca^2+^ Entry in Human Pulmonary Artery Myocytes during Proliferation. Am. J. Physiol.-Heart Circ. Physiol..

[144] Sweeney M., Yu Y., Platoshyn O., Zhang S., McDaniel S.S., Yuan J.X.-J. (2002). Inhibition of Endogenous TRP1 Decreases Capacitative Ca^2+^ Entry and Attenuates Pulmonary Artery Smooth Muscle Cell Proliferation. Am. J. Physiol.-Lung Cell. Mol. Physiol..

[145] Lin M.-J., Leung G.P.H., Zhang W.-M., Yang X.-R., Yip K.-P., Tse C.-M., Sham J.S.K. (2004). Chronic Hypoxia-Induced Upregulation of Store-Operated and Receptor-Operated Ca^2+^ Channels in Pulmonary Arterial Smooth Muscle Cells: A Novel Mechanism of Hypoxic Pulmonary Hypertension. Circ. Res..

[146] Yu Y., Fantozzi I., Remillard C.V., Landsberg J.W., Kunichika N., Platoshyn O., Tigno D.D., Thistlethwaite P.A., Rubin L.J., Yuan J.X.-J. (2004). Enhanced Expression of Transient Receptor Potential Channels in Idiopathic Pulmonary Arterial Hypertension. Proc. Natl. Acad. Sci. USA.

[147] Ng L.C., Gurney A.M. (2001). Store-Operated Channels Mediate Ca^2+^ Influx and Contraction in Rat Pulmonary Artery. Circ. Res..

[148] Yu Y., Sweeney M., Zhang S., Platoshyn O., Landsberg J., Rothman A., Yuan J.X.-J. (2003). PDGF Stimulates Pulmonary Vascular Smooth Muscle Cell Proliferation by Upregulating TRPC6 Expression. Am. J. Physiol.-Cell Physiol..

[149] McDaniel S.S., Platoshyn O., Wang J., Yu Y., Sweeney M., Krick S., Rubin L.J., Yuan J.X.-J. (2001). Capacitative Ca^2+^ Entry in Agonist-Induced Pulmonary Vasoconstriction. Am. J. Physiol.-Lung Cell. Mol. Physiol..

[150] Wang J., Shimoda L.A., Sylvester J.T. (2004). Capacitative Calcium Entry and TRPC Channel Proteins Are Expressed in Rat Distal Pulmonary Arterial Smooth Muscle. Am. J. Physiol.-Lung Cell. Mol. Physiol..

[151] Ng L.C., O’Neill K.G., French D., Airey J.A., Singer C.A., Tian H., Shen X.-M., Hume J.R. (2012). TRPC1 and Orai1 Interact with STIM1 and Mediate Capacitative Ca^2+^ Entry Caused by Acute Hypoxia in Mouse Pulmonary Arterial Smooth Muscle Cells. Am. J. Physiol.-Cell Physiol..

[152] Xia Y., Yang X.-R., Fu Z., Paudel O., Abramowitz J., Birnbaumer L., Sham J.S.K. (2014). TRPC1 and TRPC6 Contribute to Hypoxic Pulmonary Hypertension through Differential Regulation of Pulmonary Vascular Functions RR. Hypertension.

[153] Malczyk M., Veith C., Fuchs B., Hofmann K., Storch U., Schermuly R.T., Witzenrath M., Ahlbrecht K., Fecher-Trost C., Flockerzi V. (2013). Classical Transient Receptor Potential Channel 1 in Hypoxia-Induced Pulmonary Hypertension. Am. J. Respir. Crit. Care Med..

[154] Lu W., Ran P., Zhang D., Lai N., Zhong N., Wang J. (2010). Bone Morphogenetic Protein 4 Enhances Canonical Transient Receptor Potential Expression, Store-Operated Ca^2+^ Entry, and Basal [Ca^2+^]_i_ in Rat Distal Pulmonary Arterial Smooth Muscle Cells. Am. J. Physiol.-Cell Physiol..

[155] Wu J., Yu Z., Su D. (2014). BMP4 Protects Rat Pulmonary Arterial Smooth Muscle Cells from Apoptosis by PI3K/AKT/Smad1/5/8 Signaling. Int. J. Mol. Sci..

[156] Weissmann N., Dietrich A., Fuchs B., Kalwa H., Ay M., Dumitrascu R., Olschewski A., Storch U., Schnitzler M.M.Y., Ghofrani A. (2006). Classical Transient Receptor Potential Channel 6 (TRPC6) Is Essential for Hypoxic Pulmonary Vasoconstriction and Alveolar Gas Exchange. Proc. Natl. Acad. Sci. USA.

[157] Wang C., Li J.-F., Zhao L., Liu J., Wan J., Wang Y.X., Wang J., Wang C. (2009). Inhibition of SOC/Ca^2+^/NFAT Pathway Is Involved in the Anti-Proliferative Effect of Sildenafil on Pulmonary Artery Smooth Muscle Cells. Respir. Res..

[158] Ingueneau C., Huynh-Do U., Marcheix B., Athias A., Gambert P., Nègre-Salvayre A., Salvayre R., Vindis C. (2009). TRPC1 Is Regulated by Caveolin-1 and Is Involved in Oxidized LDL-induced Apoptosis of Vascular Smooth Muscle Cells. J. Cell. Mol. Med..

[159] Ng L.C., Airey J.A., Hume J.R. (2010). The Contribution of TRPC1 and STIM1 to Capacitative Ca^2+^ Entry in Pulmonary Artery. Adv. Exp. Med. Biol..

[160] Kumar B., Dreja K., Shah S.S., Cheong A., Xu S.-Z., Sukumar P., Naylor J., Forte A., Cipollaro M., McHugh D. (2006). Upregulated TRPC1 Channel in Vascular Injury In Vivo and Its Role in Human Neointimal Hyperplasia. Circ. Res..

[161] Takahashi Y., Watanabe H., Murakami M., Ono K., Munehisa Y., Koyama T., Nobori K., Iijima T., Ito H. (2007). Functional Role of Stromal Interaction Molecule 1 (STIM1) in Vascular Smooth Muscle Cells. Biochem. Biophys. Res. Commun..

[162] Li J., McKeown L., Ojelabi O., Stacey M., Foster R., O’Regan D., Porter K.E., Beech D.J. (2011). Nanomolar Potency and Selectivity of a Ca^2+^ Release-Activated Ca^2+^ Channel Inhibitor against Store-Operated Ca^2+^ Entry and Migration of Vascular Smooth Muscle Cells. Br. J. Pharmacol..

[163] Potier M., Gonzalez J.C., Motiani R.K., Abdullaev I.F., Bisaillon J.M., Singer H.A., Trebak M. (2009). Evidence for STIM1- and Orai1-Dependent Store-Operated Calcium Influx through ICRAC in Vascular Smooth Muscle Cells: Role in Proliferation and Migration. FASEB J..

[164] Guo R., Yang L., Li M., Pan X., Liu B., Deng Y. (2012). Stim1- and Orai1-Mediated Store-Operated Calcium Entry Is Critical for Angiotensin II-Induced Vascular Smooth Muscle Cell Proliferation. Cardiovasc. Res..

[165] Rodríguez-Moyano M., Díaz I., Dionisio N., Zhang X., Ávila-Medina J., Calderón-Sánchez E., Trebak M., Rosado J.A., Ordóñez A., Smani T. (2013). Urotensin-II Promotes Vascular Smooth Muscle Cell Proliferation through Store-Operated Calcium Entry and EGFR Transactivation. Cardiovasc. Res..

[166] Bisaillon J.M., Motiani R.K., Gonzalez-Cobos J.C., Potier M., Halligan K.E., Alzawahra W.F., Barroso M., Singer H.A., Jourd’heuil D., Trebak M. (2010). Essential Role for STIM1/Orai1-Mediated Calcium Influx in PDGF-Induced Smooth Muscle Migration. Am. J. Physiol.-Cell Physiol..

[167] Azimi I., Stevenson R.J., Zhang X., Meizoso-Huesca A., Xin P., Johnson M., Flanagan J.U., Chalmers S.B., Yoast R.E., Kapure J.S. (2020). A New Selective Pharmacological Enhancer of the Orai1 Ca^2+^ Channel Reveals Roles for Orai1 in Smooth and Skeletal Muscle Functions. ACS Pharmacol. Transl. Sci..

[168] Yang H., Chen X.-Y., Kuang S.-J., Zhou M.-Y., Zhang L., Zeng Z., Liu L., Wu F.-L., Zhang M.-Z., Mai L.-P. (2020). Abnormal Ca^2+^ Handling Contributes to the Impairment of Aortic Smooth Muscle Contractility in Zucker Diabetic Fatty Rats. J. Mol. Cell. Cardiol..

[169] Trebak M. (2012). STIM/Orai Signalling Complexes in Vascular Smooth Muscle. J. Physiol..

[170] Avila-Medina J., Mayoral-Gonzalez I., Dominguez-Rodriguez A., Gallardo-Castillo I., Ribas J., Ordoñez A., Rosado J.A., Smani T. (2018). The Complex Role of Store Operated Calcium Entry Pathways and Related Proteins in the Function of Cardiac, Skeletal and Vascular Smooth Muscle Cells. Front. Physiol..

[171] Kwan H.-Y., Shen B., Ma X., Kwok Y.-C., Huang Y., Man Y.-B., Yu S., Yao X. (2009). TRPC1 Associates With BKCa Channel to Form a Signal Complex in Vascular Smooth Muscle Cells. Circ. Res..

[172] Zhang S., Patel H.H., Murray F., Remillard C.V., Schach C., Thistlethwaite P.A., Insel P.A., Yuan J.X.-J. (2007). Pulmonary Artery Smooth Muscle Cells from Normal Subjects and IPAH Patients Show Divergent CAMP-Mediated Effects on TRPC Expression and Capacitative Ca^2+^ Entry. Am. J. Physiol.-Lung Cell. Mol. Physiol..

[173] He X., Song S., Ayon R.J., Balisterieri A., Black S.M., Makino A., Wier W.G., Zang W.-J., Yuan J.X.-J. (2018). Hypoxia Selectively Upregulates Cation Channels and Increases Cytosolic [Ca^2+^] in Pulmonary, but Not Coronary, Arterial Smooth Muscle Cells. Am. J. Physiol.-Cell Physiol..

[174] Bonnet S., Michelakis E.D., Porter C.J., Andrade-Navarro M.A., Thébaud B., Bonnet S., Haromy A., Harry G., Moudgil R., McMurtry M.S. (2006). An Abnormal Mitochondrial–Hypoxia Inducible Factor-1α–Kv Channel Pathway Disrupts Oxygen Sensing and Triggers Pulmonary Arterial Hypertension in Fawn Hooded Rats. Circulation.

[175] Dai Z., Li M., Wharton J., Zhu M.M., Zhao Y.-Y. (2016). Prolyl-4 Hydroxylase 2 (PHD2) Deficiency in Endothelial Cells and Hematopoietic Cells Induces Obliterative Vascular Remodeling and Severe Pulmonary Arterial Hypertension in Mice and Humans Through Hypoxia-Inducible Factor-2α. Circulation.

[176] Lei W., He Y., Shui X., Li G., Yan G., Zhang Y., Huang S., Chen C., Ding Y. (2016). Expression and Analyses of the HIF-1 Pathway in the Lungs of Humans with Pulmonary Arterial Hypertension. Mol. Med. Rep..

[177] Chen J., Sysol J.R., Singla S., Zhao S., Yamamura A., Valdez-Jasso D., Abbasi T., Shioura K.M., Sahni S., Reddy V. (2017). Nicotinamide Phosphoribosyltransferase Promotes Pulmonary Vascular Remodeling and Is a Therapeutic Target in Pulmonary Arterial Hypertension. Circulation.

[178] Lu W., Wang J., Shimoda L.A., Sylvester J.T. (2008). Differences in STIM1 and TRPC Expression in Proximal and Distal Pulmonary Arterial Smooth Muscle Are Associated with Differences in Ca^2+^ Responses to Hypoxia. Am. J. Physiol.-Lung Cell. Mol. Physiol..

[179] Giachini F.R.C., Chiao C.-W., Carneiro F.S., Lima V.V., Carneiro Z.N., Dorrance A.M., Tostes R.C., Webb R.C. (2009). CHBPR: Increased Activation of STIM-1/Orai-1 in Aorta from Hypertensive Rats: A Novel Insight into Vascular Dysfunction. Hypertension.

[180] Castillo-Galán S., Arenas G.A., Reyes R.V., Krause B.J., Iturriaga R. (2020). Stim-Activated TRPC-ORAI Channels in Pulmonary Hypertension Induced by Chronic Intermittent Hypoxia. Pulm. Circ..

[181] Castillo-Galan S., Arenas G., Krause B., Iturriaga R. (2020). Late Breaking Abstract—Contribution of STIM-Activated TRPC-ORAI Channels to the Intermittent Hypoxia-Induced Pulmonary Hypertension. Eur. Respir. J..

[182] Perros F., Montani D., Dorfmüller P., Durand-Gasselin I., Tcherakian C., Le Pavec J., Mazmanian M., Fadel E., Mussot S., Mercier O. (2008). Platelet-Derived Growth Factor Expression and Function in Idiopathic Pulmonary Arterial Hypertension. Am. J. Respir. Crit. Care Med..

[183] Schermuly R.T., Dony E., Ghofrani H.A., Pullamsetti S., Savai R., Roth M., Sydykov A., Lai Y.J., Weissmann N., Seeger W. (2005). Reversal of Experimental Pulmonary Hypertension by PDGF Inhibition. J. Clin. Investig..

[184] Balasubramaniam V., Le Cras T.D., Ivy D.D., Grover T.R., Kinsella J.P., Abman S.H. (2003). Role of Platelet-Derived Growth Factor in Vascular Remodeling during Pulmonary Hypertension in the Ovine Fetus. Am. J. Physiol.-Lung Cell. Mol. Physiol..

[185] Sun C.-K., Zhen Y.-Y., Lu H.-I., Sung P.-H., Chang L.-T., Tsai T.-H., Sheu J.-J., Chen Y.-L., Chua S., Chang H.-W. (2014). Reducing TRPC1 Expression through Liposome-Mediated SiRNA Delivery Markedly Attenuates Hypoxia-Induced Pulmonary Arterial Hypertension in a Murine Model. Stem Cells Int..

[186] Liu X.-R., Zhang M.-F., Yang N., Liu Q., Wang R.-X., Cao Y.-N., Yang X.-R., Sham J.S.K., Lin M.-J. (2012). Enhanced Store-Operated Ca^2+^ Entry and TRPC Channel Expression in Pulmonary Arteries of Monocrotaline-Induced Pulmonary Hypertensive Rats. Am. J. Physiol.-Cell Physiol..

[187] Alzoubi A., Almalouf P., Toba M., O’Neill K., Qian X., Francis M., Taylor M.S., Alexeyev M., McMurtry I.F., Oka M. (2013). TRPC4 Inactivation Confers a Survival Benefit in Severe Pulmonary Arterial Hypertension. Am. J. Pathol..

[188] Yu Y., Keller S.H., Remillard C.V., Safrina O., Nicholson A., Zhang S.L., Jiang W., Vangala N., Landsberg J.W., Wang J.-Y. (2009). A Functional Single-Nucleotide Polymorphism in the TRPC6 Gene Promoter Associated With Idiopathic Pulmonary Arterial Hypertension. Circulation.

[189] Pousada G., Baloira A., Valverde D. (2015). Molecular and Clinical Analysis of TRPC6 and AGTR1 Genes in Patients with Pulmonary Arterial Hypertension. Orphanet J. Rare Dis..

[190] Linde C.I., Karashima E., Raina H., Zulian A., Wier W.G., Hamlyn J.M., Ferrari P., Blaustein M.P., Golovina V.A. (2012). Increased Arterial Smooth Muscle Ca^2+^ Signaling, Vasoconstriction, and Myogenic Reactivity in Milan Hypertensive Rats. Am. J. Physiol. Heart Circ. Physiol..

[191] Jain P.P., Lai N., Xiong M., Chen J., Babicheva A., Zhao T., Parmisano S., Zhao M., Paquin C., Matti M. (2021). TRPC6, a Therapeutic Target for Pulmonary Hypertension. Am. J. Physiol.-Lung Cell. Mol. Physiol..

[192] Deng Z., Morse J.H., Slager S.L., Cuervo N., Moore K.J., Venetos G., Kalachikov S., Cayanis E., Fischer S.G., Barst R.J. (2000). Familial Primary Pulmonary Hypertension (Gene PPH1) Is Caused by Mutations in the Bone Morphogenetic Protein Receptor-II Gene. Am. J. Hum. Genet..

[193] Evans J.D.W., Girerd B., Montani D., Wang X.-J., Galiè N., Austin E.D., Elliott G., Asano K., Grünig E., Yan Y. (2016). BMPR2 Mutations and Survival in Pulmonary Arterial Hypertension: An Individual Participant Data Meta-Analysis. Lancet Respir. Med..

[194] Atkinson C., Stewart S., Upton P.D., Machado R., Thomson J.R., Trembath R.C., Morrell N.W. (2002). Primary Pulmonary Hypertension Is Associated with Reduced Pulmonary Vascular Expression of Type II Bone Morphogenetic Protein Receptor. Circulation.

[195] Yang X., Long L., Southwood M., Rudarakanchana N., Upton P.D., Jeffery T.K., Atkinson C., Chen H., Trembath R.C., Morrell N.W. (2005). Dysfunctional Smad Signaling Contributes to Abnormal Smooth Muscle Cell Proliferation in Familial Pulmonary Arterial Hypertension. Circ. Res..

[196] Yang J., Li X., Li Y., Southwood M., Ye L., Long L., Al-Lamki R.S., Morrell N.W. (2013). Id Proteins Are Critical Downstream Effectors of BMP Signaling in Human Pulmonary Arterial Smooth Muscle Cells. Am. J. Physiol.-Lung Cell. Mol. Physiol..

[197] Orriols M., Gomez-Puerto M.C., ten Dijke P. (2017). BMP Type II Receptor as a Therapeutic Target in Pulmonary Arterial Hypertension. Cell. Mol. Life Sci..

[198] Anderson L., Lowery J.W., Frank D.B., Novitskaya T., Jones M., Mortlock D.P., Chandler R.L., de Caestecker M.P. (2010). Bmp2 and Bmp4 Exert Opposing Effects in Hypoxic Pulmonary Hypertension. Am. J. Physiol. Regul. Integr. Comp. Physiol..

[199] Zhang Y., Lu W., Yang K., Xu L., Lai N., Tian L., Jiang Q., Duan X., Chen M., Wang J. (2013). Bone Morphogenetic Protein 2 Decreases TRPC Expression, Store-Operated Ca^2+^ Entry, and Basal [Ca^2+^]_i_ in Rat Distal Pulmonary Arterial Smooth Muscle Cells. Am. J. Physiol. Cell Physiol..

[200] Kondratska K., Kondratskyi A., Yassine M., Lemonnier L., Lepage G., Morabito A., Skryma R., Prevarskaya N. (2014). Orai1 and STIM1 Mediate SOCE and Contribute to Apoptotic Resistance of Pancreatic Adenocarcinoma. Biochim. Biophys. Acta (BBA)—Mol. Cell Res..

[201] Liang S.-J., Zeng D.-Y., Mai X.-Y., Shang J.-Y., Wu Q.-Q., Yuan J.-N., Yu B.-X., Zhou P., Zhang F.-R., Liu Y.-Y. (2016). Inhibition of Orai1 Store-Operated Calcium Channel Prevents Foam Cell Formation and Atherosclerosis. Arter. Thromb. Vasc. Biol..

[202] Vacher P., Vacher A.-M., Pineau R., Latour S., Soubeyran I., Pangault C., Tarte K., Soubeyran P., Ducret T., Bresson-Bepoldin L. (2015). Localized Store-Operated Calcium Influx Represses CD95-Dependent Apoptotic Effects of Rituximab in Non-Hodgkin B Lymphomas. J. Immunol..

[203] Weigand L., Foxson J., Wang J., Shimoda L.A., Sylvester J.T. (2005). Inhibition of Hypoxic Pulmonary Vasoconstriction by Antagonists of Store-Operated Ca^2+^ and Nonselective Cation Channels. Am. J. Physiol.-Lung Cell. Mol. Physiol..

[204] Ward J.P.T., Robertson T.P., Aaronson P.I. (2005). Capacitative Calcium Entry: A Central Role in Hypoxic Pulmonary Vasoconstriction?. Am. J. Physiol.-Lung Cell. Mol. Physiol..

[205] Shibata A., Uchida K., Kodo K., Miyauchi T., Mikoshiba K., Takahashi T., Yamagishi H. (2019). Type 2 Inositol 1,4,5-Trisphosphate Receptor Inhibits the Progression of Pulmonary Arterial Hypertension via Calcium Signaling and Apoptosis. Heart Vessels.

[206] Kiselyov K., Mignery G.A., Zhu M.X., Muallem S. (1999). The N-Terminal Domain of the IP3 Receptor Gates Store-Operated HTrp3 Channels. Mol. Cell.

[207] Tang J., Lin Y., Zhang Z., Tikunova S., Birnbaumer L., Zhu M.X. (2001). Identification of Common Binding Sites for Calmodulin and Inositol 1,4,5-Trisphosphate Receptors on the Carboxyl Termini of Trp Channels. J. Biol. Chem..

[208] Yang Z., Song T., Truong L., Reyes-García J., Wang L., Zheng Y.-M., Wang Y.-X. (2020). Important Role of Sarcoplasmic Reticulum Ca^2+^ Release via Ryanodine Receptor-2 Channel in Hypoxia-Induced Rieske Iron-Sulfur Protein-Mediated Mitochondrial Reactive Oxygen Species Generation in Pulmonary Artery Smooth Muscle Cells. Antioxid. Redox Signal..

[209] Kaßmann M., Szijártó I.A., García-Prieto C.F., Fan G., Schleifenbaum J., Anistan Y.-M., Tabeling C., Shi Y., le Noble F., Witzenrath M. (2019). Role of Ryanodine Type 2 Receptors in Elementary Ca^2+^ Signaling in Arteries and Vascular Adaptive Responses. J. Am. Heart Assoc..

[210] Gilbert G., Ducret T., Marthan R., Savineau J.-P., Quignard J.-F. (2014). Stretch-Induced Ca^2+^ Signalling in Vascular Smooth Muscle Cells Depends on Ca^2+^ Store Segregation. Cardiovasc. Res..

[211] Dahan D., Ducret T., Quignard J.-F., Marthan R., Savineau J.-P., Estève E. (2012). Implication of the Ryanodine Receptor in TRPV4-Induced Calcium Response in Pulmonary Arterial Smooth Muscle Cells from Normoxic and Chronically Hypoxic Rats. Am. J. Physiol.-Lung Cell. Mol. Physiol..

[212] Sitbon O., Humbert M., Jaïs X., Ioos V., Hamid A.M., Provencher S., Garcia G., Parent F., Hervé P., Simonneau G. (2005). Long-Term Response to Calcium Channel Blockers in Idiopathic Pulmonary Arterial Hypertension. Circulation.

[213] The Effect of High Doses of Calcium-Channel Blockers on Survival in Primary Pulmonary Hypertension|NEJM. https://www.nejm.org/doi/full/10.1056/NEJM199207093270203.

[214] He L.-P., Hewavitharana T., Soboloff J., Spassova M.A., Gill D.L. (2005). A Functional Link between Store-Operated and TRPC Channels Revealed by the 3,5-Bis(Trifluoromethyl)Pyrazole Derivative, BTP2. J. Biol. Chem..

[215] Takezawa R., Cheng H., Beck A., Ishikawa J., Launay P., Kubota H., Kinet J.-P., Fleig A., Yamada T., Penner R. (2006). A Pyrazole Derivative Potently Inhibits Lymphocyte Ca2 Influx and Cytokine Production by Facilitating Transient Receptor Potential Melastatin 4 Channel Activity. Mol. Pharmacol..

[216] Ishikawa J., Ohga K., Yoshino T., Takezawa R., Ichikawa A., Kubota H., Yamada T. (2003). A Pyrazole Derivative, YM-58483, Potently Inhibits Store-Operated Sustained Ca^2+^ Influx and IL-2 Production in T Lymphocytes. J. Immunol..

[217] Zitt C., Strauss B., Schwarz E.C., Spaeth N., Rast G., Hatzelmann A., Hoth M. (2004). Potent Inhibition of Ca^2+^ Release-Activated Ca^2+^ Channels and T-Lymphocyte Activation by the Pyrazole Derivative BTP2. J. Biol. Chem..

[218] Schleifer H., Doleschal B., Lichtenegger M., Oppenrieder R., Derler I., Frischauf I., Glasnov T., Kappe C., Romanin C., Groschner K. (2012). Novel Pyrazole Compounds for Pharmacological Discrimination between Receptor-Operated and Store-Operated Ca^2+^ Entry Pathways. Br. J. Pharmacol..

[219] Zhang X., Xin P., Yoast R.E., Emrich S.M., Johnson M.T., Pathak T., Benson J.C., Azimi I., Gill D.L., Monteith G.R. (2020). Distinct Pharmacological Profiles of ORAI1, ORAI2, and ORAI3 Channels. Cell Calcium.

[220] Derler I., Schindl R., Fritsch R., Heftberger P., Riedl M.C., Begg M., House D., Romanin C. (2013). The Action of Selective CRAC Channel Blockers Is Affected by the Orai Pore Geometry. Cell Calcium.

[221] Shawer H., Norman K., Cheng C.W., Foster R., Beech D.J., Bailey M.A. (2021). ORAI1 Ca^2+^ Channel as a Therapeutic Target in Pathological Vascular Remodelling. Front. Cell Dev. Biol..

[222] Waldherr L., Tiffner A., Mishra D., Sallinger M., Schober R., Frischauf I., Schmidt T., Handl V., Sagmeister P., Köckinger M. (2020). Blockage of Store-Operated Ca^2+^ Influx by Synta66 Is Mediated by Direct Inhibition of the Ca^2+^ Selective Orai1 Pore. Cancers.

[223] Ng S.W., di Capite J., Singaravelu K., Parekh A.B. (2008). Sustained Activation of the Tyrosine Kinase Syk by Antigen in Mast Cells Requires Local Ca^2+^ Influx through Ca^2+^ Release-Activated Ca^2+^ Channels. J. Biol. Chem..

[224] Sabatino A.D., Rovedatti L., Kaur R., Spencer J.P., Brown J.T., Morisset V.D., Biancheri P., Leakey N.A.B., Wilde J.I., Scott L. (2009). Targeting Gut T Cell Ca^2+^ Release-Activated Ca^2+^ Channels Inhibits T Cell Cytokine Production and T-Box Transcription Factor T-Bet in Inflammatory Bowel Disease. J. Immunol..

[225] Bartoli F., Bailey M.A., Rode B., Mateo P., Antigny F., Bedouet K., Gerbaud P., Gosain R., Plante J., Norman K. (2020). Orai1 Channel Inhibition Preserves Left Ventricular Systolic Function and Normal Ca ^2+^ Handling After Pressure Overload. Circulation.

[226] Trebak M., Earley S. (2018). Signal Transduction and Smooth Muscle.

[227] Sadaghiani A.M., Lee S.M., Odegaard J.I., Leveson-Gower D.B., McPherson O.M., Novick P., Kim M.R., Koehler A.N., Negrin R., Dolmetsch R.E. (2014). Identification of Orai1 Channel Inhibitors by Using Minimal Functional Domains to Screen Small Molecule Microarrays. Chem. Biol..

[228] Kim K.-D., Srikanth S., Tan Y.-V., Yee K., Jew M., Damoiseaux R., Jung M., Shimizu S., An D., Ribalet B. (2013). Calcium Signaling via Orai1 Is Essential for Induction of the Nuclear Orphan Receptor Pathway To Drive Th17 Differentiation. J. Immunol..

[229] Smyth J.T., DeHaven W.I., Bird G.S., Putney J.W. (2008). Ca^2+^-Store-Dependent and -Independent Reversal of Stim1 Localization and Function. J. Cell Sci..

[230] Kiyonaka S., Kato K., Nishida M., Mio K., Numaga T., Sawaguchi Y., Yoshida T., Wakamori M., Mori E., Numata T. (2009). Selective and Direct Inhibition of TRPC3 Channels Underlies Biological Activities of a Pyrazole Compound. Proc. Natl. Acad. Sci. USA.

[231] Miller M., Shi J., Zhu Y., Kustov M., Tian J., Stevens A., Wu M., Xu J., Long S., Yang P. (2011). Identification of ML204, a Novel Potent Antagonist That Selectively Modulates Native TRPC4/C5 Ion Channels. J. Biol. Chem..

[232] Bauer C.C., Minard A., Pickles I.B., Simmons K.J., Chuntharpursat-Bon E., Burnham M.P., Kapur N., Beech D.J., Muench S.P., Wright M.H. (2020). Xanthine-Based Photoaffinity Probes Allow Assessment of Ligand Engagement by TRPC5 Channels. RSC Chem. Biol..

[233] Just S., Chenard B.L., Ceci A., Strassmaier T., Chong J.A., Blair N.T., Gallaschun R.J., del Camino D., Cantin S., D’Amours M. (2018). Treatment with HC-070, a Potent Inhibitor of TRPC4 and TRPC5, Leads to Anxiolytic and Antidepressant Effects in Mice. PLoS ONE.

[234] Rubaiy H.N., Ludlow M.J., Henrot M., Gaunt H.J., Miteva K., Cheung S.Y., Tanahashi Y., Hamzah N., Musialowski K.E., Blythe N.M. (2017). Picomolar, Selective, and Subtype-Specific Small-Molecule Inhibition of TRPC1/4/5 Channels. J. Biol. Chem..

[235] Minard A., Bauer C.C., Wright D.J., Rubaiy H.N., Muraki K., Beech D.J., Bon R.S. (2018). Remarkable Progress with Small-Molecule Modulation of TRPC1/4/5 Channels: Implications for Understanding the Channels in Health and Disease. Cells.

[236] Yu M., Ledeboer M.W., Daniels M., Malojcic G., Tibbitts T.T., Coeffet-Le Gal M., Pan-Zhou X.-R., Westerling-Bui A., Beconi M., Reilly J.F. (2019). Discovery of a Potent and Selective TRPC5 Inhibitor, Efficacious in a Focal Segmental Glomerulosclerosis Model. ACS Med. Chem. Lett..

[237] Vinayagam D., Quentin D., Yu-Strzelczyk J., Sitsel O., Merino F., Stabrin M., Hofnagel O., Yu M., Ledeboer M.W., Nagel G. (2020). Structural Basis of TRPC4 Regulation by Calmodulin and Pharmacological Agents. eLife.

[238] Maier T., Follmann M., Hessler G., Kleemann H.-W., Hachtel S., Fuchs B., Weissmann N., Linz W., Schmidt T., Löhn M. (2015). Discovery and Pharmacological Characterization of a Novel Potent Inhibitor of Diacylglycerol-Sensitive TRPC Cation Channels. Br. J. Pharmacol..

[239] Merritt J.E., Armstrong W.P., Benham C.D., Hallam T.J., Jacob R., Jaxa-Chamiec A., Leigh B.K., McCarthy S.A., Moores K.E., Rink T.J. (1990). SK&F 96365, a Novel Inhibitor of Receptor-Mediated Calcium Entry. Biochem. J..

[240] Washburn D.G., Holt D.A., Dodson J., McAtee J.J., Terrell L.R., Barton L., Manns S., Waszkiewicz A., Pritchard C., Gillie D.J. (2013). The Discovery of Potent Blockers of the Canonical Transient Receptor Channels, TRPC3 and TRPC6, Based on an Anilino-Thiazole Pharmacophore. Bioorg. Med. Chem. Lett..

[241] Seo K., Rainer P.P., Shalkey Hahn V., Lee D., Jo S.-H., Andersen A., Liu T., Xu X., Willette R.N., Lepore J.J. (2014). Combined TRPC3 and TRPC6 Blockade by Selective Small-Molecule or Genetic Deletion Inhibits Pathological Cardiac Hypertrophy. Proc. Natl. Acad. Sci. USA.

[242] Lin B.L., Matera D., Doerner J.F., Zheng N., del Camino D., Mishra S., Bian H., Zeveleva S., Zhen X., Blair N.T. (2019). In Vivo Selective Inhibition of TRPC6 by Antagonist BI 749327 Ameliorates Fibrosis and Dysfunction in Cardiac and Renal Disease. Proc. Natl. Acad. Sci. USA.

[243] Häfner S., Burg F., Kannler M., Urban N., Mayer P., Dietrich A., Trauner D., Broichhagen J., Schaefer M. (2018). A (+)-Larixol Congener with High Affinity and Subtype Selectivity toward TRPC6. ChemMedChem.

[244] Bai Y., Yu X., Chen H., Horne D., White R., Wu X., Lee P., Gu Y., Ghimire-Rijal S., Lin D.C.-H. (2020). Structural Basis for Pharmacological Modulation of the TRPC6 Channel. eLife.

[245] Wen L., Voronina S., Javed M.A., Awais M., Szatmary P., Latawiec D., Chvanov M., Collier D., Huang W., Barrett J. (2015). Inhibitors of ORAI1 Prevent Cytosolic Calcium-Associated Injury of Human Pancreatic Acinar Cells and Acute Pancreatitis in 3 Mouse Models. Gastroenterology.

[246] Bruen C., Miller J., Wilburn J., Mackey C., Bollen T.L., Stauderman K., Hebbar S. (2021). Auxora for the Treatment of Patients With Acute Pancreatitis and Accompanying Systemic Inflammatory Response Syndrome. Pancreas.

[247] Miller J., Bruen C., Schnaus M., Zhang J., Ali S., Lind A., Stoecker Z., Stauderman K., Hebbar S. (2020). Auxora versus Standard of Care for the Treatment of Severe or Critical COVID-19 Pneumonia: Results from a Randomized Controlled Trial. Crit. Care.

[248] Barde P.J., Viswanadha S., Veeraraghavan S., Vakkalanka S.V., Nair A. (2021). A First-in-Human Study to Evaluate the Safety, Tolerability and Pharmacokinetics of RP3128, an Oral Calcium Release-Activated Calcium (CRAC) Channel Modulator in Healthy Volunteers. J. Clin. Pharm. Ther..

[249] PRCL Research Inc A Phase 2a Study to Evaluate Safety, Tolerability, and Efficacy of PRCL-02 in Patients with Moderate to Severe Chronic Plaque Psoriasis; Clinicaltrials.gov: 2020.

[250] Walsh L., Reilly J.F., Cornwall C., Gaich G.A., Gipson D.S., Heerspink H.J.L., Johnson L., Trachtman H., Tuttle K.R., Farag Y.M.K. (2021). Safety and Efficacy of GFB-887, a TRPC5 Channel Inhibitor, in Patients With Focal Segmental Glomerulosclerosis, Treatment-Resistant Minimal Change Disease, or Diabetic Nephropathy: TRACTION-2 Trial Design. Kidney Int. Rep..

[251] Li J., Zhang X., Song X., Liu R., Zhang J., Li Z. (2019). The Structure of TRPC Ion Channels. Cell Calcium.

[252] Zhao Y., McVeigh B.M., Moiseenkova-Bell V.Y. (2021). Structural Pharmacology of TRP Channels. J. Mol. Biol..

